# Alteration of replication protein A binding mode on single-stranded DNA by NSMF potentiates RPA phosphorylation by ATR kinase

**DOI:** 10.1093/nar/gkad543

**Published:** 2023-06-28

**Authors:** Yujin Kang, Ye Gi Han, Keon Woo Khim, Woo Gyun Choi, Min Kyung Ju, Kibeom Park, Kyeong Jin Shin, Young Chan Chae, Jang Hyun Choi, Hongtae Kim, Ja Yil Lee

**Affiliations:** Department of Biological Sciences, Ulsan National Institute of Science and Technology, Ulsan 44919, Republic of Korea; Department of Biological Sciences, Ulsan National Institute of Science and Technology, Ulsan 44919, Republic of Korea; Department of Biological Sciences, Ulsan National Institute of Science and Technology, Ulsan 44919, Republic of Korea; Department of Biological Sciences, Ulsan National Institute of Science and Technology, Ulsan 44919, Republic of Korea; Department of Biological Sciences, Ulsan National Institute of Science and Technology, Ulsan 44919, Republic of Korea; Department of Biological Sciences, Ulsan National Institute of Science and Technology, Ulsan 44919, Republic of Korea; Department of Biological Sciences, Ulsan National Institute of Science and Technology, Ulsan 44919, Republic of Korea; Department of Biological Sciences, Ulsan National Institute of Science and Technology, Ulsan 44919, Republic of Korea; Department of Biological Sciences, Ulsan National Institute of Science and Technology, Ulsan 44919, Republic of Korea; Institute of Basic Science Center for Genomic Integrity, Ulsan 44919, Republic of Korea; Department of Biological Sciences, Ulsan National Institute of Science and Technology, Ulsan 44919, Republic of Korea; Institute of Basic Science Center for Genomic Integrity, Ulsan 44919, Republic of Korea; Department of Biological Sciences, Ulsan National Institute of Science and Technology, Ulsan 44919, Republic of Korea; Institute of Basic Science Center for Genomic Integrity, Ulsan 44919, Republic of Korea

## Abstract

Replication protein A (RPA), a eukaryotic single-stranded DNA (ssDNA) binding protein, dynamically interacts with ssDNA in different binding modes and plays essential roles in DNA metabolism such as replication, repair, and recombination. RPA accumulation on ssDNA due to replication stress triggers the DNA damage response (DDR) by activating the ataxia telangiectasia and RAD3-related (ATR) kinase, which phosphorylates itself and downstream DDR factors, including RPA. We recently reported that the N-methyl-D-aspartate receptor synaptonuclear signaling and neuronal migration factor (NSMF), a neuronal protein associated with Kallmann syndrome, promotes RPA32 phosphorylation via ATR upon replication stress. However, how NSMF enhances ATR-mediated RPA32 phosphorylation remains elusive. Here, we demonstrate that NSMF colocalizes and physically interacts with RPA at DNA damage sites *in vivo* and *in vitro*. Using purified RPA and NSMF in biochemical and single-molecule assays, we find that NSMF selectively displaces RPA in the more weakly bound 8- and 20-nucleotide binding modes from ssDNA, allowing the retention of more stable RPA molecules in the 30-nt binding mode. The 30-nt binding mode of RPA enhances RPA32 phosphorylation by ATR, and phosphorylated RPA becomes stabilized on ssDNA. Our findings provide new mechanistic insight into how NSMF facilitates the role of RPA in the ATR pathway.

## Graphical Abstract

**Graphical Abstract:**
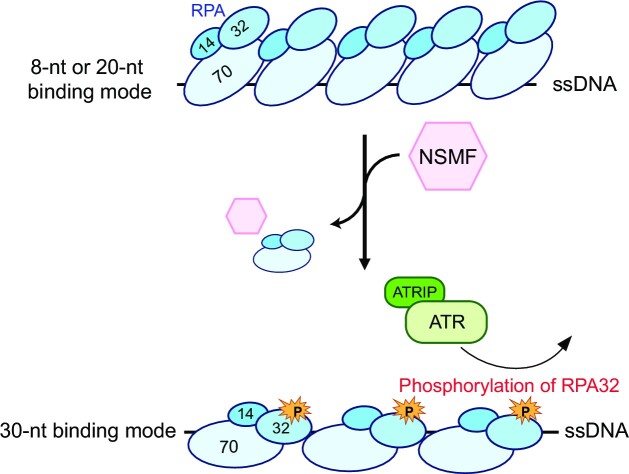

## INTRODUCTION

The eukaryotic single-stranded DNA-binding protein RPA interacts dynamically with ssDNA as a heterotrimeric complex consisting of RPA70, RPA32, and RPA14. RPA plays important roles in diverse DNA metabolic reactions, such as replication, repair, and recombination, by protecting transient ssDNA and preventing secondary structure formation of ssDNA (1,2). DNA-bound RPA interacts with many proteins to guide processes in DNA metabolism. RPA binding to ssDNA is primarily an intermediate stage in DNA metabolism. In addition, RPA-coated ssDNA provides a platform for the DNA damage response (DDR) induced by replication stress (3–5). When replication forks encounter DNA lesions, such as inter-strand crosslinks (ICLs), pyrimidine dimers, or bulky lesions, they can stall, leading to the uncoupling of DNA helicase and DNA polymerase and the generation of ssDNA covered with RPA. Long RPA–ssDNA filaments trigger the DDR primarily by activating an apical kinase, the ataxia telangiectasia and RAD3-related (ATR) kinase, through a complex with ATR-interacting protein (ATRIP) (5,6). The Rad17–RFC2–5 clamp loader then loads the Rad9–Rad1–Hus1 (9–1–1) complex on the recessed 5′ terminus of the nascent primer downstream of the stalled primer-template junction, leading to the stimulation of ATR kinase activity aided by TopBP1 (7–11). Additionally, the RPA–ssDNA complex can be bound by ETAA1 to activate ATR kinase (12). Finally, Chk1 is activated by phosphorylation by ATR, leading to cell-cycle arrest, DNA repair, and replication fork stabilization (13). During this process, ATR-ATRIP phosphorylates RPA and RPA phosphorylation is essential for recruiting downstream proteins to the stalled fork and activating ATR signaling. Moreover, RPA phosphorylation may minimize ssDNA generation under replication stress by stimulating repair DNA synthesis (14,15). Despite the importance of RPA phosphorylation, the molecular details of ATR-mediated RPA phosphorylation are poorly understood.

Central to the DNA-binding activity of RPA are DNA-binding oligonucleotide/oligosaccharide-binding folds (here referred to as DNA-binding domains [DBDs]). RPA70 has four DBDs: DBD-A, DBD-B, DBD-C, and DBD-F, while RPA32 and RPA14 have DBD-D and DBD-E, respectively (1,2,16–18). RPA binds ssDNA in at least three distinct binding modes defined by the length of the ssDNA stretches directly bound to RPA (16). The lowest affinity 8-nucleotide (nt) mode has a binding affinity (*K*_d_) of approximately 100 nM, in which only DBD-A and DBD-B of RPA70 bind to ssDNA (19,20). The second mode is the ∼20-nt binding mode with a *K*_d_ of about 5 nM, in which DBD-A, DBD-B, and DBD-C of RPA70 bind ssDNA (21–23). The third mode is the highest affinity 30-nt binding mode with a *K*_d_ of approximately 0.05 nM, in which DBD-A, DBD-B, and DBD-C of RPA70 and DBD-D of RPA32 are all engaged with ssDNA (18,19). The binding modes can change dynamically (23,24). RPA must tightly bind to ssDNA to accomplish its biological function; however, it must also be displaced to allow access to downstream proteins. Therefore, the dynamic behavior of RPA and the various binding modes may be important for DNA metabolism.

*N*-methyl-d-aspartate receptor synaptonuclear signaling and neuronal migration factor (NSMF), also known as Jacob (the mouse ortholog is NELF [nasal embryonic LHRH factor]), is a 60 kDa neuronal protein originally identified as being expressed in both central and peripheral nervous system tissues during embryonic development (25,26). It is involved in the migration of olfactory and GnRH (gonadotropin-releasing hormone) neurons into the forebrain and is related to neuronal plasticity (25,26). Defects in NSMF are associated with Kallmann syndrome, which is characterized by an impaired sense of smell (anosmia) and idiopathic hypogonadotropic hypogonadism (IHH) (27–29). In addition, NSMF carries the extracellular signal-regulated kinase (ERK) phosphorylation signal to the nucleus in response to synaptic and extrasynaptic NMDA receptor stimulation (28,30). Although NSMF is highly expressed in the brain, it is also weakly expressed in other organs (e.g. heart, liver, testis, and kidney) (26,29). Moreover, NSMF is expressed in myoblast skeletal muscle cells and may enhance myoblast proliferation (31). We recently observed that NSMF is involved in the DDR pathway and that NSMF deficiency increases the sensitivity to DNA damage (32). As a scaffold, NSMF, along with cell division cycle 5-like (CDC5L) and ATR-ATRIP, enhances RPA32 phosphorylation. However, how NSMF mediates DDR is unknown. In this study, we investigated the molecular mechanism underlying how NSMF activates ATR-mediated RPA32 phosphorylation. We observed that NSMF is recruited to DNA damage sites at a very early stage and interacts with RPA. Using purified NSMF and RPA in single-molecule and biochemical assays, we demonstrated that NSMF promotes the more stable 30-nt binding mode of RPA on ssDNA to facilitate RPA32 phosphorylation and stabilization.

## MATERIALS AND METHODS

All the details of the materials and methods are described in the Supplementary Data. Here, they are briefly described.

### Cell-based experiments

*Cell culture*. HeLa and human embryonic kidney (HEK) 293T cells were purchased from the American Type Culture Collection (ATCC). The cell lines were maintained in Dulbecco's modified Eagle's medium (DMEM) (Invitrogen) supplemented with 10% fetal bovine serum (FBS) (Millipore) and 1% penicillin/streptomycin (Gibco) at 37°C with 5% CO_2_. The NSMF knockout (KO) cell line was cultured as described previously (32). For chromatin fraction analysis, HeLa cells stably expressing FLAG-NSMF-wild-type (WT) or ΔD2 were obtained upon antibiotic selection with 3 μg/ml puromycin (InvivoGen) for 2 weeks. Clones were pooled into a single population to avoid clonal heterogeneity.

*Laser microirradiation and imaging of cells*. Laser microirradiation was performed with a Nikon A1 laser microdissection system equipped with a 37°C chamber and CO_2_ module (Nikon, Tokyo, Japan). An ultraviolet A laser with a 355 nm wavelength was illuminated, and time-lapse fluorescence images were acquired at 10 or 15-s intervals.

*Transfection and small interfering RNAs (siRNAs)*. Transient transfection of plasmid DNA and siRNAs was performed using Lipofectamine 3000 (Thermo Fisher Scientific) and Lipofectamine RNAiMAX (Thermo Fisher Scientific). The control siRNAs were previously described (33). The following custom siRNA sequences, RPA32 #1: 5′-GGCUCCAACCAACAUUGUU-3′ and RPA32 #2: 5′-CCUAGUUUCACAAUCUGUU-3′, were synthesized by Bioneer Inc. (South Korea).

*Plasmids*. The SFB-NSMF, FLAG-NSMF, GFP-NSMF, the NSMF D-1, D-2, D-3, D-4 and D-5 deletion mutants, and Myc-RPA32 expression plasmids were described previously (32). All other plasmid constructs are described in the Supplementary Data.

### Immunoprecipitation

Detailed immunoprecipitation materials and methods are provided in the Supplementary Data.

### Chromatin fraction analysis

NSMF KO HeLa cells were treated with or without 2 mM hydroxyurea (HU) for 16 hrs. The cells were then collected and washed with 1×PBS. The collected cells were lysed in NETN buffer at 4°C for 30 min. The crude lysates were cleared by centrifugation at 13 000 rpm for 10 min at 4°C, and the pellet was resuspended in 0.2 M HCl for 1 h. The resuspended mixture was centrifuged at 13 000 rpm for 10 min at 4°C. The supernatant chromatin fraction was neutralized with 1 M Tris–HCl [8.0] and mixed with 2×SDS sample buffer (0.15 M Tris–HCl [6.8], 10% beta-mercaptoethanol, 1.2% SDS, 30% glycerol and 0.04% bromophenol blue) and boiled at 95°C for 10 min. Histone 3 (H3) served as a control for the chromatin fraction. To see the effect of NSMF-ΔD2 on RPA32 phosphorylation in the presence of HU, NSMF KO HeLa cells were complemented with stable FLAG-NSMF-WT or ΔD2 expression. Chromatin fraction analysis was conducted as described above.

### Protein purification

All details for the protein purification are described in the Supplementary Data. All proteins, including NSMF, RPA, each RPA subunit, and RPA mutants, were purified from proper *E. coli* strains, and purification was performed at 4°C. Briefly, NSMF and the NSMF-ΔD2 mutant were cloned into a pET19b-derived plasmid containing maltose binding protein (MBP), a PreScission cleavage site, 3×FLAG at the amino (N)-terminus, and 10×His at the carboxyl (C)-terminus (Figure S2A). The proteins were purified with an amylose resin, and the MBP-tag was cleaved out using the PreScission protease (27–0843-01, Cytiva). The eluant was further purified by gel filtration using Superdex200 10/300 GL (17-5175-01, Cytiva) and finally stored at -80°C after dialysis. Human RPA and its derivatives were purified as previously described with modifications (34). Purified *Escherichia coli* single-stranded binding (EcSSB) was purchased (S3917, Sigma Aldrich).

### DNA preparation

Oligomers were synthesized (Bioneer, South Korea) and are listed in the Supplementary Table. All DNA constructs were prepared as previously described (35). The oligomers were mixed at equimolar ratios in 10 mM Tris–HCl [7.5] and 100 mM NaCl. For annealing, the mixtures were heated at 95°C for 10 min and slowly cooled to 23°C.

### Biochemical and biophysical assays

All the details for the biochemical assays are described in the Supplementary Data.

*Electrophoretic mobility shift assay (EMSA)*. All reactions were performed in reaction buffer (50 mM Tris–HCl [7.5], 50 mM NaCl, 1 mM DTT, and 0.01% Tween-20) at 23°C. Fluorescently labeled ssDNA (10 nM) or other DNA constructs were used (Supplementary Table) for NSMF binding or the RPA/NSMF titration. The gels were imaged using the Typhoon RGB (GE Healthcare) and analyzed by ImageJ (NIH).

*Magnetic bead pulldown assay*. Biotinylated ssDNA was conjugated to streptavidin-coated magnetic beads (Dynabeads M-280, Invitrogen). RPA (1.2 μM) was bound to the ssDNA-decorated magnetic beads, and then NSMF or NSMF-ΔD2 was incubated with the beads in reaction buffer at 23°C for 30 min. After boiling the reactants at 95°C for 3 min, the supernatant and eluant were analyzed by western blotting.

*Single-molecule photobleaching assay*. The single-molecule photobleaching assay was conducted as described previously (36). All reactions were performed at 23°C in reaction buffer. Biotinylated ssDNA was anchored on a streptavidin-coated slide and then incubated with RPA-eGFP (Supplementary Table). In the presence of NSMF or NSMF-ΔD2 mutant, the fluorescence signal from RPA-eGFP was collected by illumination with a 488 nm laser until almost all eGFP fluorescent puncta were photobleached. Fluorescence signals were analyzed with customized software.

*In vitro phosphorylation of RPA*. *In vitro* phosphorylation was performed using a previous protocol with minor modifications (37,38). Purified RPA was phosphorylated with 2 ng/μl pcDNA3, 2 ng/μl M13mp18 ssDNA (NEB, N4040S) and 10 mg/ml HEK293T cell lysate in 1×phosphorylation buffer (40 mM HEPES [7.5], 8 mM MgCl_2_, 3 mM ATP, and 0.5 mM DTT). RPA was further purified with a Ni-NTA resin (HisPur™ Ni-NTA Resin. Thermo Fisher Scientific, # 88222). The proteins were concentrated using Amicon with buffer exchange into storage buffer (25 mM Tris–HCl [7.5], 40 mM NaCl, 1 mM DTT, 0.5 mM EDTA and 10% glycerol).

*In vitro* binding assay. Purified RPA and eGFP-tagged NSMF-WT or ΔD2 were incubated in NETN buffer at 4°C for 1 h. Anti-GFP-coupled protein G agarose (Thermo Fisher Scientific, Pierce Protein G Agarose, 20399) was then added, and the samples were incubated at 4°C for an additional 1 h. The immunoprecipitates were washed three times with NETN buffer and analyzed by western blotting.

*ATR kinase assay*. The details for the ATR kinase assays are described in the Supplementary Data. Briefly, 10-nt, 20-nt, and 30-nt ssDNA oligomers (dT_10_, dT_20_, and dT_30_) were synthesized by Bioneer (South Korea) (Supplementary Table). Human ATR protein was pulled down from crude extracts of HeLa cells treated with 2 mM HU for 16 hrs using protein A-agarose (CA-PRI-0005, Repligen) conjugated to ATR antibodies (A300-137A, Bethyl Laboratories). For the 30-nt binding mode effect, 75 nM RPA was incubated with 30 nM ssDNA (dT_10_, dT_20_, or dT_30_) in ATR kinase buffer (20 mM HEPES [8.0], 10 mM MgCl_2_, 2 mM DTT, and 0.1 mM ATP) at 23°C for 20 min. The reactant (25 μl) was added to the immunoprecipitated ATR kinase and incubated at 30°C for 30 min. For the NSMF effect, excess RPA (150 nM) was incubated with 10 nM 91-nt ssDNA in ATR kinase buffer at 23°C for 20 min, and then the samples were incubated with 80 nM NSMF for 30 min. The reactant (25 μl) was added to the immunoprecipitated ATR kinase and incubated at 30°C for 30 min. After the reactions, all samples were separated by SDS-PAGE and analyzed by western blotting with the indicated antibodies.

## RESULTS

### NSMF is recruited to DNA damage sites and interacts with RPA both *in vivo* and *in vitro*

**Figure 1.**
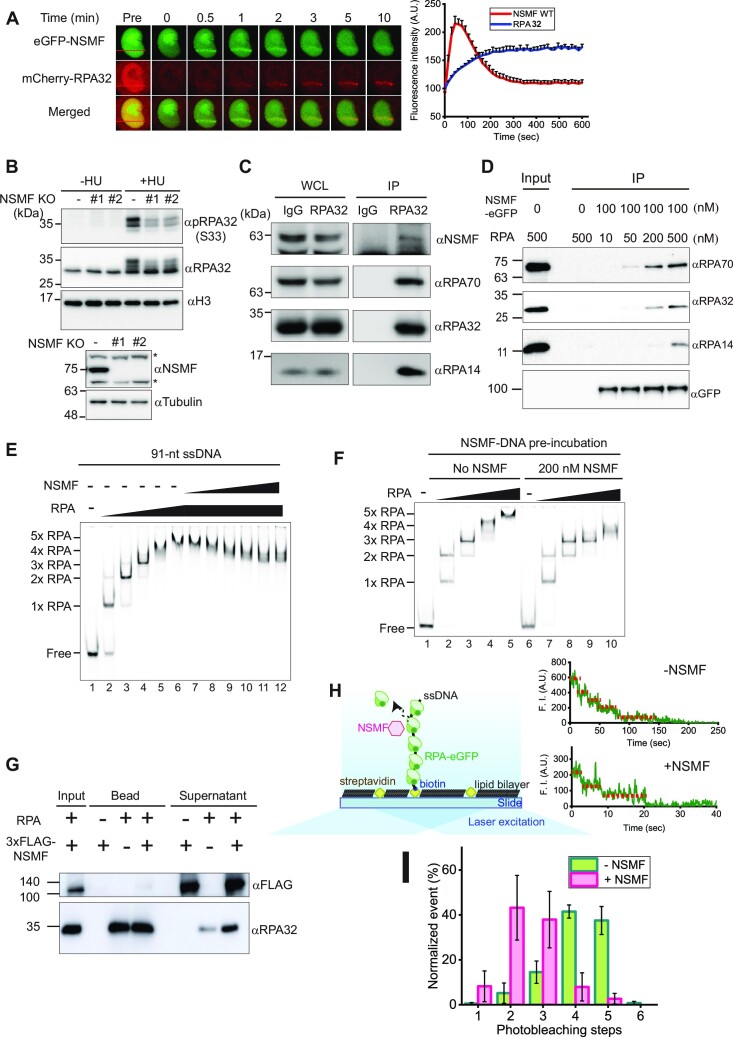
NSMF interacts with RPA at DNA damage sites and dissociates RPA from ssDNA. (**A**) Laser microirradiation experiments were performed on HeLa cells transfected with eGFP-NSMF and mCherry-RPA32. The kinetics of NSMF and RPA32 localization at DNA damage sites were examined. Left: fluorescence images of a laser-irradiated cell as a function of time. The red lines indicate the laser-irradiated sites. Right: quantification of relative fluorescence intensity of eGFP and mCherry. The initial fluorescent intensity at the damage site was set as 100 for each cell, and the recruitment kinetics were plotted. The intensity was averaged for more than 10 cells under each condition, and the data represent the mean + SEM for three repeated experiments. (**B**) (Top) Chromatin fraction analysis for the NSMF effect on RPA32 phosphorylation under replication stress. (Bottom) NSMF expression in WT and KO cells. The asterisks (*) represent nonspecific bands. (**C**) Endogenous immunoprecipitation (IP) assay for NSMF and the RPA trimer in HeLa cells. WCL: whole cell lysates. (**D**) *In vitro* IP assay for NSMF and RPA binding. Purified NSMF and RPA trimer were immunoprecipitated with anti-GFP at different concentrations. Each RPA subunit was analyzed by western blotting. (**E**) EMSA for NSMF and the RPA–ssDNA complex. RPA (0, 25, 75, 100, 125, and 150 nM) was titrated with 10 nM 91-nt ssDNA. NSMF (0, 10, 20, 40, 80, 140, and 200 nM) was titrated with the RPA (150 nM)–ssDNA complex. (**F**) The effect of NSMF on the formation of RPA–ssDNA complexes. Left: only RPA (0, 25, 75, 125, and 150 nM) was titrated with the 91-nt ssDNA without NSMF. Right: NSMF (200 nM) was pre-incubated with 10 nM ssDNA, and then RPA was titrated. (**G**) Magnetic bead pulldown assay for RPA dissociation by NSMF. The supernatant and bound fractions were analyzed by western blotting using an anti-FLAG antibody (αFLAG) for NSMF and an RPA32 antibody (αRPA32). (**H**) Single-molecule photobleaching assay for NSMF-mediated RPA dissociation from ssDNA. Left: schematic of the single-molecule photobleaching assay. The 91-nt ssDNA was anchored on a slide surface passivated by a lipid bilayer via a biotin-streptavidin linkage. eGFP molecules tagged on RPA proteins were bleached step-wise under continuous laser illumination, and the fluorescence signal was detected by total internal reflection fluorescence microscopy. Right: time traces of the fluorescence intensities of RPA-eGFP bound to ssDNA molecules in the absence (top) or presence (bottom) of NSMF. The steps of fluorescence intensity indicate the photobleaching events of individual eGFP molecules. (**I**) Histogram for the photobleaching steps of RPA-eGFPs bound to ssDNA in the presence (magenta) or absence (green) of NSMF. Error bars represent the standard deviation in triplicate. The number of analyzed molecules was >100.

Previously, we reported that NSMF regulates RPA32 phosphorylation and ubiquitination in response to replication stress (32). To investigate the interaction between NSMF and RPA for RPA32 phosphorylation in the DDR pathway, we conducted laser microirradiation experiments using HeLa cells (Figure 1A). NSMF was localized to laser-induced DNA damage sites at very early time points (less than 3 min) and subsequently dissipated, while RPA gradually accumulated at the same sites but with slower kinetics. In NSMF KO cells, RPA translocated to the DNA damage sites with similar kinetics as in the wild-type (WT) cells (Figure S1A). Conversely, in RPA32 knockdown (KD) cells, NSMF was recruited to the DNA damage sites as it was in WT cells (Figure S1B). These results showed that NSMF and RPA do not modulate each other's recruitment to DNA damage sites.

We also determined the effect of NSMF on RPA32 phosphorylation under replication stress using the chromatin fraction assay with NSMF-WT and KO HeLa cells (Figure 1B). HU treatment resulted in RPA32 serine 33 (S33) phosphorylation, which was compromised in two independently derived NSMF KO cell lines, confirming that NSMF enhances RPA32 phosphorylation in the presence of replication stress. We next examined the endogenous binding between NSMF and RPA by immunoprecipitation (IP) using HeLa cell extracts and found that NSMF and other RPA subunits were immunoprecipitated with RPA32 (Figure 1C). Moreover, we also tested the binding between NSMF and RPA using an overexpression system (Figure S1C and D). In this IP assay, NSMF interacted with RPA regardless of benzonase, suggesting that NSMF binds directly to RPA. FLAG-NSMF was immunoprecipitated from HEK293T cell lysates using anti-FLAG beads. RPA70 and RPA14 co-precipitated with NSMF, whereas only a weak signal was observed for RPA32. Taken together, our IP data suggested that NSMF and RPA interact in cells.

To directly probe the interaction between NSMF and RPA using purified proteins, we expressed and purified full-length NSMF tagged with MBP and 3×FLAG at the N-terminus and eGFP at the C-terminus from *E. coli* (Figure S2A). MBP was cleaved off using the HRV 3C protease. We also purified the RPA trimer consisting of the RPA70, RPA32, and RPA14 subunits. In addition, we purified eGFP-tagged RPA trimer and individual RPA32 and RPA14 subunits. All RPA constructs were expressed in *E. coli* (Figure S2B). The purified RPA70 subunit was purchased. We investigated the direct binding between NSMF and RPA using the purified proteins by IP and found that all RPA subunits were co-immunoprecipitated with NSMF (Figure 1D). In contrast, when NSMF was incubated with each individual RPA subunit, none of them were immunoprecipitated with NSMF, indicating that NSMF interacts with the trimeric complex of RPA (Figure S2C).

Next, we tested whether NSMF could bind to DNA using EMSA with the various nucleic acid constructs (Figure S3A, B). NSMF bound to ssDNA, primer-template junctions (PTJ), and fork structure (Y-shape) with low affinity. No binding was detected for duplex, bubble, D-loop, ssDNA gap, and R-loop structures. Our EMSA data suggested that NSMF preferentially binds to oligonucleotides containing a free ssDNA end with low affinity.

### NSMF mediates the rearrangement of RPA to a more stable binding mode on ssDNA

We investigated the interaction between NSMF and RPA–ssDNA complexes using EMSA. For the 91-nt ssDNA, as the RPA concentration increased, the bands shifted to yield protein-DNA complexes with five distinct mobilities, which we interpreted to be 1–5 RPA molecules per ssDNA (Figure 1E). To see the effect of NSMF on RPA bound to ssDNA, RPA was incubated with the 91-nt ssDNA, and then NSMF was added to the reaction. Surprisingly, the addition of increasing amounts of NSMF to the reaction at the highest RPA concentration (150 nM) resulted in RPA-DNA bands with increased mobility (Figure 1E). The band shift also occurred when NSMF was pre-incubated with ssDNA prior to the addition of RPA, further indicating that NSMF could modulate RPA binding to ssDNA (Figure 1F). To test whether NSMF had a similar effect on a single-stranded binding protein (SSB) from a different species, we used *E. coli* SSB (EcSSB), which has three distinct binding modes (35-nt, 56-nt, and 65-nt) depending on the salt concentration (39–41). At the salt concentration used in our RPA experiments (50 mM NaCl), the three modes can coexist, and combinations of the modes are possible depending on the length of the ssDNA. We tested 91-nt ssDNA (Figure S2D). As low EcSSB concentrations, one band shift appeared, and this band transitioned to a slower migrating band at higher concentrations. This indicates that the 91-nt ssDNA accommodates two EcSSB molecules in other binding modes; presumably one in the 35-nt mode and the other in the 56-nt binding mode. When the saturated EcSSB-ssDNA complexes were titrated with NSMF, the bands did not change. Our data showed that in the presence of NSMF, no transitions between binding modes between EcSSBs were observed, suggesting that the effect of NSMF on the mobility of the RPA–DNA complexes was species-specific. Finally, we tested whether NSMF remained on the RPA–ssDNA complex using EMSA with NSMF-eGFP and Cy5-labeled ssDNA to which unlabeled RPA binds. NSMF-eGFP did not colocalize with Cy5-ssDNA but remained in the well, indicating that NSMF did not co-bind with the RPA–ssDNA complex (Figure S3D).

To test whether NSMF actively dissociated RPA from ssDNA, we performed magnetic bead pulldown assays. NSMF was added to RPA-bound 91-nt ssDNA immobilized on magnetic beads *via* a biotin-streptavidin linkage. Analysis of the supernatant fractions revealed that more RPA molecules were dissociated from the ssDNA in the presence of NSMF, suggesting that NSMF promoted the dissociation of RPA from the ssDNA (Figure 1G). RPA dissociation from ssDNA was quantified using the single-molecule photobleaching assay (Figure 1H). RPA-eGFP was bound to ssDNA anchored on a slide surface. The number of RPA molecules bound to ssDNA was estimated by counting the photobleaching events of individual eGFP fluorophores tagged on RPA under continuous laser illumination. In the absence of NSMF, most of the ssDNA was bound by four to five RPA molecules. The observation of two and four photobleaching steps in the presence and absence of NSMF, respectively, likely results from non-fluorescence or inactive eGFP, which has been observed in similar applications (42,43). The addition of NSMF reduced the number of bound RPA molecules to two or three per ssDNA (Figure 1I). The single-molecule photobleaching data quantitatively demonstrated the rearrangement of RPA to a more stable binding mode in the presence of NSMF, presumably by releasing less stably bound RPA molecules. These data are consistent with the EMSA results in Figure 1E. Taken together, our data suggested that NSMF promotes the rearrangement of RPA to bind ssDNA in a more favorable binding mode.

### NSMF deletion mutant lacking the RPA binding domain does not destabilize RPA from ssDNA

**Figure 2.**
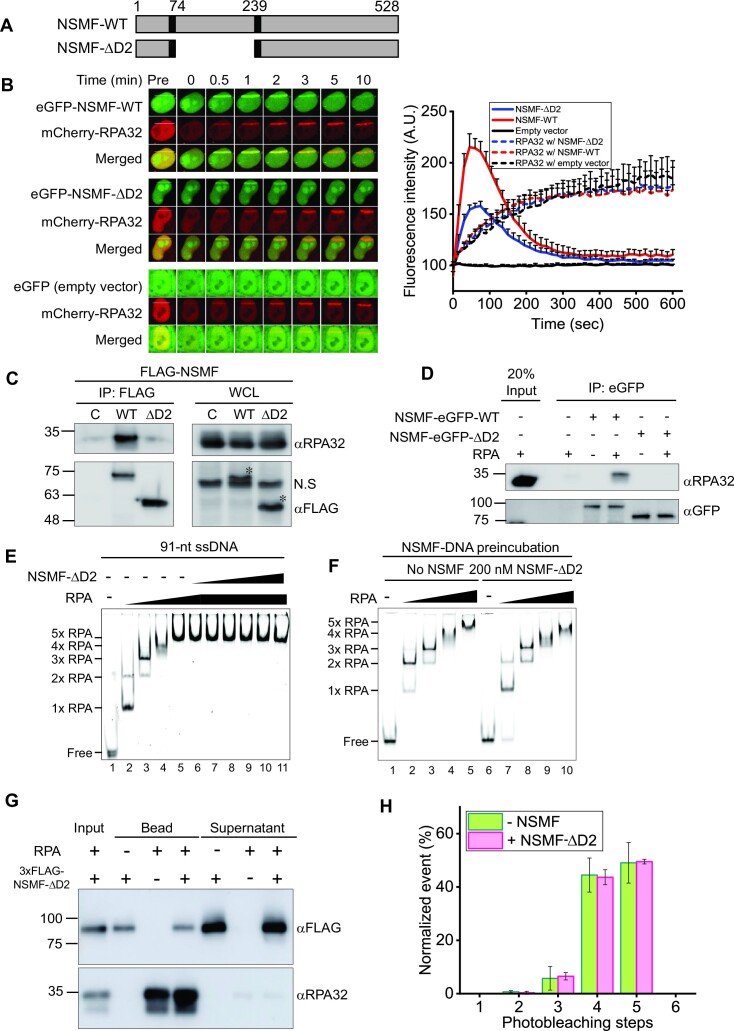
RPA binding–defective mutant NSMF-ΔD2 does not destabilize RPA from ssDNA. (**A**) Domain constructs for NSMF-WT and NSMF-ΔD2, a mutant in which RPA interaction domain D2 (74–239 amino acids) was deleted. (**B**) The laser microirradiation experiments were performed with NSMF KO HeLa cells transfected with mCherry-RPA32 and eGFP-NSMF WT, ΔD2, or empty vector. Left: fluorescent images of laser-irradiated cells as a function of time. The white lines indicate the laser-irradiated sites. Right: quantification of the relative fluorescence intensities of eGFP and mCherry. The initial fluorescent intensity at the damage site was set as 100 for each cell, and the recruitment kinetics were plotted. The average intensities for >10 cells for each condition are presented. Data represent the mean + SEM for three repeated experiments. (**C**) Interaction between RPA32 and either NSMF-WT or NSMF-ΔD2 was determined using immunoprecipitation from HeLa cells transfected with FLAG-NSMF WT or ΔD2. The asterisks denoted bands for NSMF WT and ΔD2. (**D**) *In vitro* binding assay using purified RPA32 and either purified NSMF-eGFP WT or ΔD2. (**E**) EMSA for the NSMF-ΔD2 and RPA–ssDNA complex. RPA (0, 25, 75, 125, and 150 nM) was titrated with 10 nM 91-nt ssDNA, and NSMF-ΔD2 (0, 10, 20, 40, 80, 140, and 200 nM) was titrated with the RPA–ssDNA complex at 150 nM RPA. (**F**) The effect of NSMF-ΔD2 on the formation of the RPA–ssDNA complex. Left: only RPA was titrated with 91-nt ssDNA without NSMF. Right: NSMF-ΔD2 (200 nM) was pre-incubated with 10 nM ssDNA and then titrated with RPA (0, 25, 75, 125, and 150 nM). (**G**) Magnetic bead pulldown assay for RPA dissociation by NSMF-ΔD2. NSMF-ΔD2 was incubated with RPA–ssDNA complexes coated on magnetic beads. The supernatant and bound fractions were analyzed by western blotting using an anti-FLAG antibody (αFLAG) for NSMF and an anti-RPA32 antibody (αRPA32). (**H**) Histogram for the photobleaching steps of RPA-eGFP bound to ssDNA in the presence (magenta) or absence (green) of NSMF-ΔD2. Error bars represent the standard deviation in triplicate. The number of analyzed molecules was >100.

We previously reported that NSMF interacts with RPA through the D2 domain (amino acids 74–239) (Figure 2A) (32). We explored the effect of the D2 domain on RPA recruitment to DNA damage sites. We conducted laser microirradiation experiments with HeLa cells transfected with eGFP-NSMF-WT, a D2 domain deletion mutant (eGFP-NSMF-ΔD2), or an empty vector together with a mCherry-RPA32 expressing vector (Figure 2B). Although less NSMF-ΔD2 accumulated at sites of DNA damage than NSMF-WT, neither NSMF-WT nor NSMF-ΔD2 altered the accumulation of RPA32 at these sites. We also investigated the effect of NSMF-ΔD2 on RPA32 phosphorylation upon replication stress using chromatin fraction analysis (Figure S4A). In response to HU treatment, RPA32 phosphorylation was reduced when NSMF-ΔD2, but not WT NSMF, was expressed in NSMF KO cells.

We next tested the binding of NSMF-ΔD2 to RPA using IP. RPA did not interact with NSMF-ΔD2 overexpressed in HeLa cells (Figures 2C and S4B), and we did not detect any interactions between purified NSMF-ΔD2 and RPA (Figures 2D and S4C). Next, we determined whether NSMF-ΔD2 could modulate the binding of RPA to ssDNA using EMSA. We observed that NSMF-ΔD2 did not alter the binding of RPA to the 91-nt ssDNA (Figure 2E). Similarly, incubation of NSMF-ΔD2 with ssDNA prior to the RPA addition did not change the EMSA results (Figure 2F). Finally, NSMF-ΔD2 did not promote RPA dissociation from ssDNA in the magnetic bead pulldown (Figure 2G) or single-molecule photobleaching (Figure 2H) assays. Thus, our data suggested that the interaction between RPA and NSMF is dependent on the D2 domain and that this specific interaction is critical for modifying the interaction of RPA with ssDNA.

### NSMF promotes the stable 30-nt binding mode of RPA by destabilizing the 8-nt and 20-nt binding modes

**Figure 3.**
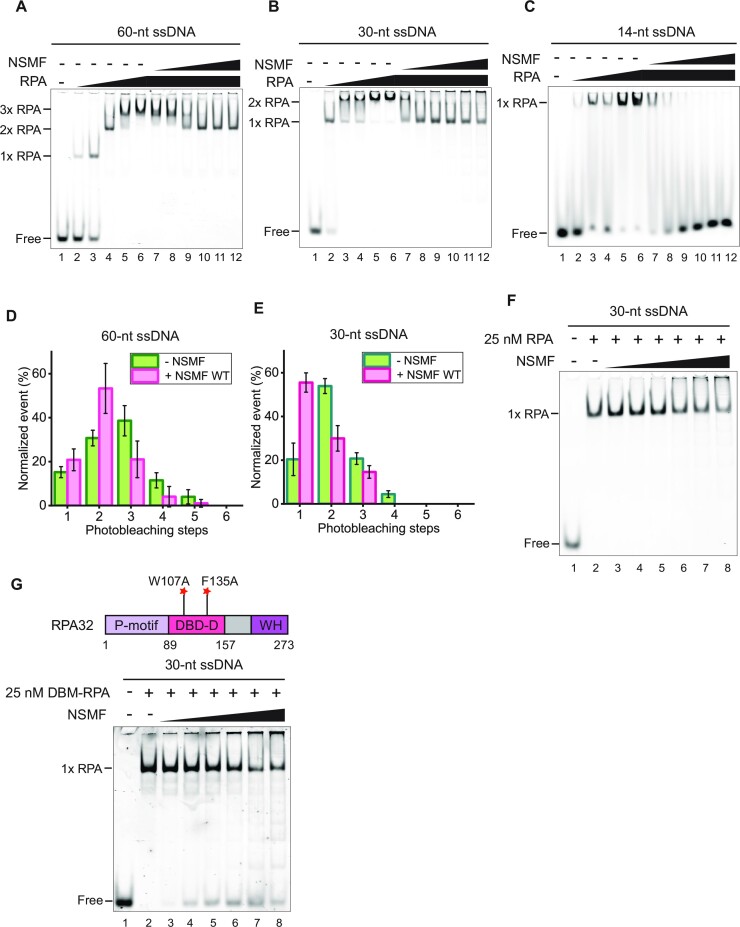
NSMF modulates the RPA binding mode. (A–C) EMSA for NSMF with RPA bound to (**A**) 60-nt, (**B**) 30-nt, and (**C**) 14-nt ssDNA. RPA (0, 25, 75, 100, 125, and 150 nM) was titrated with 10 nM of each ssDNA. RPA (150 nM) was titrated with NSMF (0, 10, 20, 40, 80, 140, and 200 nM). (D, E) Histograms for photobleaching events of RPA-eGFP bound to (**D**) 60-nt and (**E**) 30-nt ssDNA in the presence (magenta) or absence (green) of NSMF. The addition of NSMF reduced the number of bound RPA molecules. More than 100 molecules were analyzed for each dataset. Error bars represent the standard deviation in triplicate. (**F**) NSMF (0, 10, 20, 40, 80, 140, and 200 nM) was titrated with 25 nM RPA bound to 30-nt ssDNA. At 25 nM, RPA had a single binding mode (30-nt mode) without a band shift. (**G**) Top: the domain structure for the DNA binding defective mutant of RPA (DBM-RPA), in which Trp107 and Phe135 were both replaced by Ala. Bottom: EMSA for NSMF with DBM-RPA and 30-nt ssDNA. DBM-RPA (25 nM) was bound to 10 nM 30-nt ssDNA and then titrated with NSMF (0, 10, 20, 40, 80, 140, and 200 nM).

RPA dynamically binds to ssDNA in different binding modes (16,19–23,44–46). Our EMSA and single-molecule photobleaching results demonstrated that multiple RPA molecules bind to ssDNA in a step-wise manner, with up to at least five RPA molecules associated with the 91-nt ssDNA at high protein concentrations. With five RPA molecules per 91 nucleotides, not all RPA molecules can bind in the most stable 30-nt binding mode (Figure 1E, I). Although the addition of NSMF altered the binding mode to the preferred 30-nt mode for three RPA molecules, excess NSMF could not remove RPA from the ssDNA (Figure 1E). Based on these results, we assumed that NSMF partially disrupts RPA molecules in the 8-nt and 20-nt binding modes and promotes more stable RPA binding on ssDNA in the 30-nt binding mode. To test whether NSMF could alter the RPA binding modes, we conducted EMSA using ssDNA of various lengths (Figure 3A–C). For the 60-nt and 30-nt ssDNA, RPA titration resulted in the appearance of three and two distinct RPA–ssDNA complexes, respectively. The addition of NSMF to both complexes caused the disappearance of the band shift with the lowest mobility, suggesting that unstably bound RPA molecules were released (Figure 3A, B).

We quantitatively estimated the number of RPA molecules bound to ssDNA using single-molecule photobleaching assays. In the absence of NSMF, three and two bleaching steps were predominant for the 60-nt and 30-nt ssDNA molecules, respectively, suggesting that the RPA molecules were associated with ssDNA in modes other than the 30-nt binding mode (Figure 3D, E). In the presence of NSMF, the overall number of bleaching steps was reduced by one, consistent with the EMSA results. Two RPA molecules per 60-nt ssDNA and one RPA molecule per 30-nt ssDNA are consistent with the 30-nt binding mode of RPA and strongly support our model that NSMF has the ability to remove RPA molecules bound to ssDNA in the 8-nt or 20-nt binding modes and could rearrange the binding mode of the remaining RPA molecules to the more stable 30-nt mode. In support of this hypothesis, we found that RPA bound to 14-nt ssDNA, for which only the 8-nt binding mode is possible as indicated by a single shifted band, was completely released by NSMF (Figure 3C). Likewise, in the single-molecule photobleaching assay with the 14-nt ssDNA, almost all fluorescent puncta disappeared when NSMF was added, indicating that NSMF completely dissociated single RPA-eGFP in the 8-nt binding mode from the 14-nt ssDNA (Figure S5A, B). These EMSA and single-molecule photobleaching experiments demonstrated that NSMF dislodges RPA molecules associated with ssDNA when they are in the less stable 8-nt and 20-nt binding modes and promotes the more stable 30-nt binding mode of RPA on ssDNA. In contrast, NSMF-ΔD2 could not detach RPA from ssDNA even on short ssDNAs (Figure S5C–G). Consistent with this model, NSMF was unable to alter the binding of RPA bound to 30-nt ssDNA, for which only a single band was observed by EMSA. Even at high concentrations of NSMF, the mobility of the RPA–ssDNA band was unaffected (Figure 3F).

### NSMF more severely destabilizes RPA with DNA binding–defective RPA32

Our results suggested that NSMF dissociated unstably bound RPA from ssDNA, promoting the stable 30-nt mode. We, therefore, hypothesized that NSMF would destabilize the ssDNA binding of an RPA mutant defective in the 30-nt binding mode. The DBD-D domain of RPA32 specifically engages with ssDNA in the 30-nt binding mode (18,19). Thus, we determined the effect of NSMF on the DNA binding of an RPA32 mutant (DBM-RPA), in which Trp107 and Phe135 were both replaced with Ala (Figure 3G). DBM-RPA weakly binds to ssDNA and suppresses the 30-nt binding mode (46,47). Accordingly, laser microirradiation experiments with DBM-RPA showed that the recruitment of DBM-RPA to DNA damage sites was impaired (Figure S6A). At 25 nM, a single DBM-RPA molecule bound to 30-nt ssDNA. However, when EMSA was performed in the presence of increasing amounts of NSMF, the free DNA band reappeared, indicating that DBM-RPA dissociated from ssDNA due to NSMF (Figure 3G). These results support our model that NSMF can dissociate ssDNA-bound low-affinity forms of RPA.

### Direct NSMF-RPA interactions are needed for the dissociation of RPA from ssDNA

**Figure 4.**
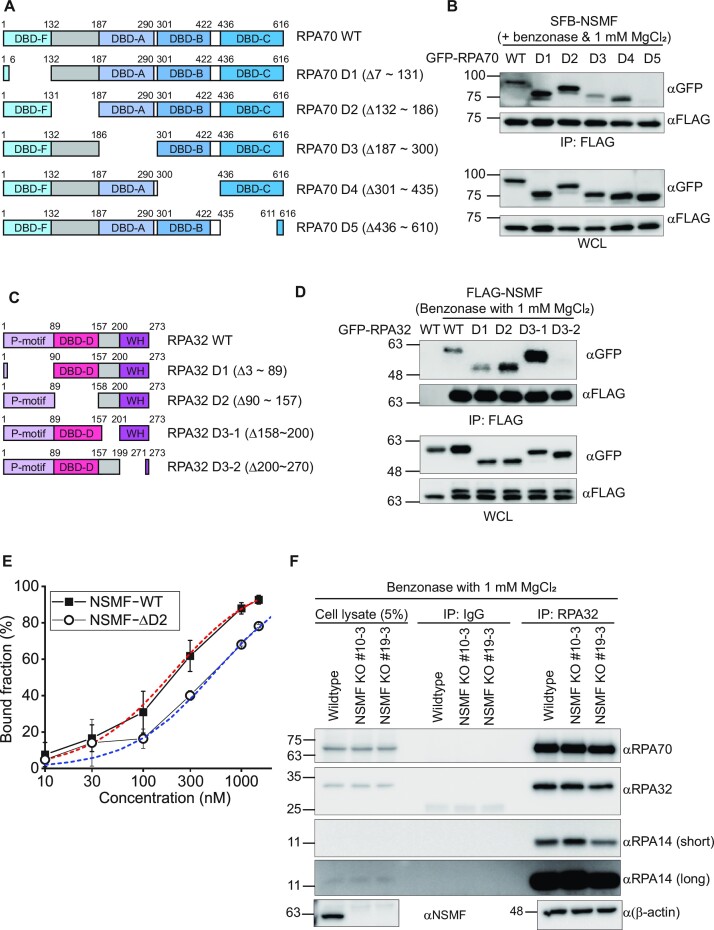
RPA destabilization mechanism by NSMF. (**A**) Domain constructs for the RPA70 deletion mutants. (**B**) IP assays between NSMF and the RPA70 deletion mutants in the presence of benzonase. NSMF interacted with the DBD-C domain (D5: 436–610 amino acids). (**C**) Domain constructs for the RPA32 deletion mutants. (**D**) IP assays between NSMF and the RPA32 deletion mutants in the presence of benzonase. NSMF interacted with the C-terminal domain (D3-2: 200–270 amino acids). (**E**) Quantification of the EMSA data for NSMF (WT or ΔD2) and ssDNA in Figure S3A and S3C. Filled square: NSMF-WT and blank circle: NSMF-ΔD2. The error bars represent the standard deviation in triplicate. The quantified data were fitted using the hyperbola function, $100( \% )[ {NSMF} ]/( {{K_d} + [ {NSMF} ]} )$. The *K*_d_ values for NSMF-WT and NSMF-ΔD2 on ssDNA were estimated to be 200 ± 22 nM and 540 ± 59 nM, respectively. (**F**) Endogenous RPA70 and RPA14 in NSMF-WT or KO HeLa cell lysates were immunoprecipitated with an anti-RPA32 antibody, and western blotting was performed using the indicated antibodies. NSMF did not disrupt the RPA trimer in the absence of DNA *in vivo*.

Given the data showing that NSMF could destabilize DBM-RPA, we hypothesized that RPA detachment by NSMF results from NSMF-mediated disruption of DNA binding by RPA70. We investigated which regions of RPA70 and RPA32 were responsible for the interaction of RPA with NSMF; the RPA14 subunit was too small to divide into subdomains. We performed IP assays with NSMF and RPA in which individual domains of RPA70 and RPA32 were deleted. We found that the deletion of the DBD-C domain in RPA70 strongly impaired the interaction between NSMF and RPA, and the deletion of the DBD-A domain only weakly disrupted the interaction with NSMF, indicating that RPA70-DBD-C was the major determinant for NSMF binding; RPA70-DBD-A had a minor role in this binding (Figures 4A, B, and S6B). The interaction was confirmed by add-back mutants (Figure S6C, D). For RPA32, deleting the C-terminal winged-helix (WH) domain impaired its interaction with NSMF (Figures 4C, D and S6E). We also tested the possibility that NSMF and RPA compete for binding to ssDNA. Using EMSA, we estimated the binding affinities (*K*_d_) of NSMF-WT and NSMF-ΔD2 for ssDNA (Figures S3A, C, and 4E). The *K*_d_ value of NSMF-WT was 200 ± 22 nM, which was comparable to that of the 8-nt binding mode (*K*_d_ = ∼100 nM) and hence could compete for binding to ssDNA, whereas the *K*_d_ of NSMF-ΔD2 was 540 ± 59 nM, about five times higher than that of the 8-nt binding mode of RPA. Thus, the mutant was not an efficient competitor for binding (Figure 4E). Finally, we examined whether NSMF could disrupt the RPA trimeric complex. RPA70 and RPA14 were co-immunoprecipitated with RPA32 in NSMF-proficient and -deficient cells, suggesting that NSMF does not disrupt the formation of the RPA trimer *in vivo* (Figure 4F). Taken together, our data suggested that a direct interaction between NSMF and RPA and the competition for ssDNA binding between NSMF and RPA contribute to the dissociation of the 8- and 20-nt binding forms of RPA from ssDNA.

### RPA phosphorylation by ATR is enhanced in the stable 30-nt binding mode *in vitro*

**Figure 5.**
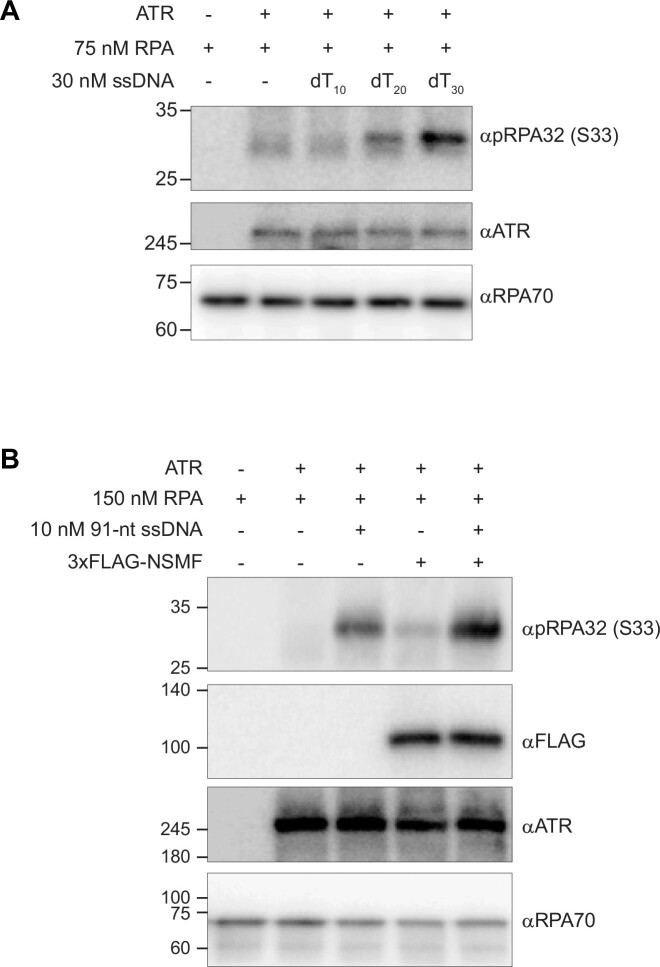
RPA 30-nt binding mode induced by NSMF enhances RPA32 phosphorylation by ATR *invitro*. (**A**) *In vitro* phosphorylation of RPA32 by ATR depends on the ssDNA length (10-nt [dT_10_], 20-nt [dT_20_], or 30-nt [dT_30_] ssDNA consisting of only thymines). ATR was pulled down from HeLa cell extracts treated with 2 mM hydroxyurea using anti-ATR antibody-coated beads. The samples were analyzed by western blotting using the indicated antibodies. (**B**) NSMF-mediated enhancement of RPA32 phosphorylation by ATR *in vitro*. Excess RPA was incubated with 91-nt ssDNA followed by 80 nM NSMF and pulled-down ATR. The proteins were analyzed by western blotting using the indicated antibodies.

We examined whether the RPA binding modes influence RPA32 phosphorylation by ATR *in vitro* using ATR isolated with anti-ATR antibody-coated beads. We tested different lengths of ssDNA (dT_10_, dT_20_, and dT_30_) to which RPA binds in a different binding mode. RPA phosphorylation was dramatically enhanced with 30-nt ssDNA (Figure 5A). Because only 30-nt ssDNA provides the stable 30-nt binding mode of RPA, our result demonstrated that the stable 30-nt binding mode enhances RPA32 phosphorylation. Next, using 91-nt ssDNA with excess RPA, we investigated whether NSMF could enhance RPA32 phosphorylation by ATR *in vitro*. As shown in Figure 5A, the presence of 91-nt ssDNA increased RPA32 phosphorylation. When NSMF was added, RPA32 phosphorylation was increased further, indicating that NSMF enhances RPA32 phosphorylation (Figure 5B). These results demonstrated that NSMF mediates the 30-nt binding mode of RPA, enhancing RPA32 phosphorylation.

### NSMF does not destabilize phosphorylated RPA from ssDNA

**Figure 6.**
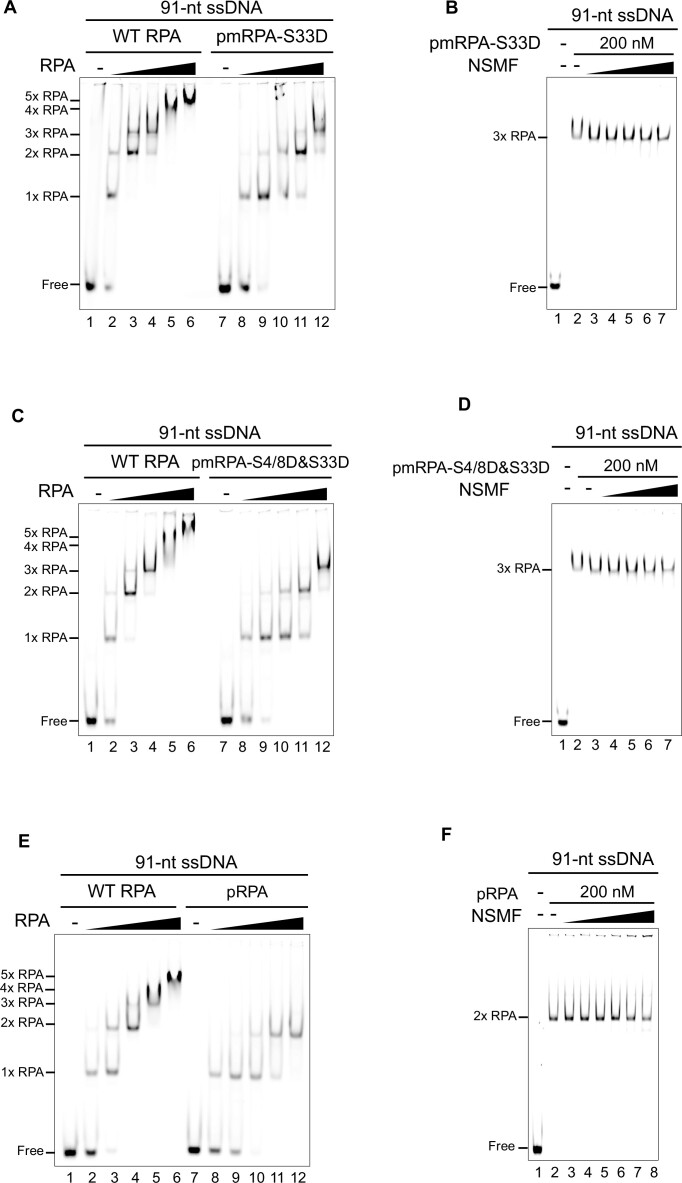
NSMF does not destabilize phosphorylated RPA. (**A**) EMSA for the binding of WT RPA and pmRPA-S33D (0, 25, 75, 100, 125, and 150 nM) to 91-nt ssDNA. (**B**) EMSA for the NSMF effect on pmRPA-S33D. NSMF (0, 20, 40, 80, 140, and 200 nM) was titrated with 200 nM pmRPA-S33D pre-incubated with 10 nM 91-nt ssDNA. (**C**) EMSA for WT RPA and pmRPA-S4/8D&S33D (0, 25, 75, 100, 125, and 150 nM) to compare their binding to 91-nt ssDNA. (**D**) EMSA for the NSMF effect on pmRPA-S4/8D&S33D. NSMF (0, 20, 40, 80, 140, and 200 nM) was titrated with 200 nM pmRPA-S4/8D&S33D pre-incubated with 10 nM 91-nt ssDNA. (**E**) EMSA for WT RPA and pRPA (0, 25, 75, 100, 125, and 150 nM) to compare their binding to 91-nt ssDNA. (**F**) EMSA for NSMF effect on pRPA. NSMF (0, 10, 20, 40, 80, 140, and 200 nM) was titrated with 200 nM pRPA pre-incubated with 10 nM 91-nt ssDNA.

We hypothesized that one of the roles of the RPA rearrangement on ssDNA is to facilitate phosphorylation in the DDR. To test whether the phosphorylation status of RPA affects its interaction with NSMF, we investigated the interaction between NSMF and a phosphomimetic derivative of RPA (pmRPA), where S33, the main ATR phosphorylation site upon replication stress, is replaced by Asp (D) (pmRPA-S33D) (48,49). Unlike WT RPA, which displayed five distinct RPA–ssDNA bands in the EMSA, only three bands were observed for pmRPA-S33D binding to 91-nt ssDNA (Figure 6A), suggesting that pmRPA-S33D prefers the most stable 30-nt binding mode even in the absence of NSMF. Consistent with this idea, when NSMF was added, there was no apparent change in the mobility of the RPA–ssDNA complexes, even at high NSMF concentrations (Figure 6B). For the 60-nt and 30-nt ssDNAs, pmRPA-S33D titration resulted in two- and one-step band shifts, respectively, which was a different pattern from WT RPA (Figure S7A, C). Like 91-nt ssDNA, NSMF did not dissociate pmRPA-S33D from 60-nt and 30-nt ssDNA (Figure S7B, D). The three-, two-, and one-step shifts for the 91-nt, 60-nt, and 30-nt ssDNA, respectively, indicated that pmRPA-S33D bound to ssDNA mostly in the 30-nt mode. Therefore, we concluded that NSMF did not rearrange the pmRPA-S33D binding mode for ssDNA because pmRPA-S33D was already stably bound to ssDNA in the 30-nt mode. In contrast, pmRPA-S33D was released from the 14-nt ssDNA similarly to WT RPA because it could not bind to this substrate in the 30-nt binding mode (Figure S7E, F).

After S33 of RPA32 is phosphorylated by ATR, the N-terminus of RPA32 is further phosphorylated by additional kinases at Ser4 (S4) and Ser8 (S8) (48–50). We examined the interaction between NSMF and multi-phosphorylated RPA using a phosphomimetic triple mutant (pmRPA-S4/8D&S33D). Like pmRPA-S33D, pmRPA-S4/8D&S33D bound to ssDNA in the 30-nt binding mode and was resistant to dissociation by NSMF (Figures 6C, D and S8A–D), suggesting that additional phosphorylation events do not affect the 30-nt binding mode. For the 14-nt ssDNA, NSMF could release pmRPA-S4/8&33D from ssDNA similar to pmRPA-S33D (Figure S8E, F).

To corroborate our findings, we directly phosphorylated RPA *in vitro* using the SV40 replication system in HEK293T cell extracts (37,38). Although the number of phosphorylated residues could not be accurately determined, incubation with phospho-specific antibodies revealed that at least S33 and S4/8 were phosphorylated (Figure S9A). We examined the effect of RPA phosphorylation on ssDNA binding and its interaction with NSMF. Like the phosphomimetics, RPA phosphorylated *in vitro* (pRPA) caused triple-, double-, and single-step shifts for the 91-nt, 60-nt, and 30-nt ssDNA, respectively, which were not rearranged upon NSMF addition (Figures 6E, F and S9B–E). Interestingly, pRPA did not bind to the 14-nt ssDNA, suggesting that the phosphorylation of RPA32 lowers the binding affinity of the DBD-A and B domains (Figure S9F, G).

## DISCUSSION

The accumulation of RPA on ssDNA in response to replication stress is needed for ATR activation, which results in the phosphorylation of ATR itself, RPA, and downstream proteins, such as Chk1 (3,5). RPA phosphorylation is in turn essential for recruiting repair enzymes to stalled replication forks. We previously reported that a deficiency in NSMF reduces RPA phosphorylation upon replication stress (32). In the current work, we demonstrate that NSMF facilitates RPA phosphorylation by rearranging RPA binding to ssDNA to the most stable 30-nt binding mode.

### The mode of RPA binding to ssDNA is concentration-dependent

It is well-known that RPA dynamically binds to ssDNA in three distinct modes that are classified by the length of ssDNA occluded by RPA: the 8-nt binding mode (*K*_d_ ≈ 100 nM), the 20-nt binding mode (*K*_d_ ≈ 5 nM), and the 30-nt binding mode (*K*_d_ ≈ 0.05 nM) (16,19,21–23,45). Employing EMSA and single-molecule photobleaching assays with different lengths of ssDNA, we observed that multiple RPA molecules bound to ssDNA. Because the 30-nt binding mode provides the highest binding affinity, we expected that the number of RPA molecules bound to the ssDNA oligomers would be restricted by the 30-nt mode. However, at higher RPA concentrations, it became evident that more than one RPA molecule per 30 nucleotides can occupy ssDNA, such that not all RPA molecules bind in the 30-nt binding mode. Our results are consistent with a previous study reporting that the DNA binding of RPA is compacted at high RPA concentrations (22). We do not know the binding modes of RPA molecules in the compact conformation on ssDNA. All RPA molecules could bind in the less stable modes, or some molecules could associate in the 30-nt mode and others in the less stable modes.

### NSMF changes RPA binding to ssDNA to the most stable mode

We found that NSMF releases RPAs bound in the less stable modes from ssDNA and promotes the most stable 30-nt binding mode of RPA. The transition from the 8-nt to 30-nt mode accompanies conformational changes in the RPA70-DBD-A/B domains and ssDNA and the sequential association of RPA70-DBD-C and RPA32-DBD-D, which form a compact Tri-C structure along with DBD-E (18,51). We showed that NSMF binds preferentially to the RPA70-DBD-C domain and the RPA32 C-terminal WH domain (Figures S2C, 4B, D, and S6B–E). In particular, the NSMF D2 domain (74 to 239 amino acids) physically interacts with RPA70 and RPA32 and is essential for RPA release (Figures 2C, D and S4B, C). The WH domain of RPA32 interacts with several proteins, including Rad52, XPA, and SV40 T antigen (16,52,53). In particular, the interaction between the SV40 T antigen and the WH domain may play a role in displacing RPA from ssDNA for SV40 replication activity (54). It is likely that the interaction between NSMF and the WH domain contributes to the RPA displacement from ssDNA. This hypothesis is supported by the evidence that NSMF destabilizes the ssDNA binding of DBM-RPA, which is defective in the DNA binding of RPA32 (Figure 3G). In addition, the binding affinity of NSMF to ssDNA is comparable to that of the 8-nt binding mode of human RPA (Figure 4E). We, therefore, propose that NSMF releases RPA from ssDNA by disrupting the binding of DBD-C or Tri-C to ssDNA and competing with RPA for ssDNA. When RPA molecules are partially dissociated from ssDNA, the remaining RPA molecules can occupy the vacant ssDNA and become stabilized in the 30-nt binding mode. The rearrangement of RPA binding modes by NSMF can occur by passive or active model. In the passive model, NSMF only detaches RPA molecules in the less stable modes, and RPA adopts the stable 30-nt binding mode upon the displacement of the other binding modes. Alternatively, NSMF may actively rearrange RPA to the 30-nt binding mode after the displacement of other binding modes. In our study, we cannot discern the two models. On the other hand, once RPA is stabilized in the 30-nt binding mode, NSMF no longer disturbs RPA binding (Figure 3F).

### The 30-nt binding mode enhances RPA32 phosphorylation

Our *in vitro* ATR kinase assays demonstrated that the 30-nt binding mode increases RPA32 phosphorylation (Figure 5A). A previous study using DNA-PK reported that RPA32 phosphorylation was increased for the 30-nt binding mode (55). In the 30-nt binding mode, the DBD-D of RPA32 binds to ssDNA, stabilizing RPA binding (16). These results suggest that the engagement of DBD-D with ssDNA is important for RPA32 phosphorylation. In addition, RPA32 phosphorylation by ATR was increased by NSMF in the presence of excess RPA (Figure 5B). Taken together, our results showed that NSMF rearranges RPA into the 30-nt binding mode, enhancing RPA32 phosphorylation by ATR.

### NSMF does not destabilize phosphorylated RPA

We also investigated how NSMF acts on RPA after ATR phosphorylates RPA32. S33 of RPA32 is the primary phosphorylation target of ATR in response to replication stress (48,49). RPA32 is hyperphosphorylated via the ATR pathway (50). To determine the consequence of RPA phosphorylation, we first tested the ssDNA binding of the phosphomimetic RPA derivative pmRPA-S33D. Unlike WT, pmRPA-S33D did not bind ssDNA in the compact, less stable binding modes; it only bound ssDNA in the 30-nt binding mode. Even though the number of bound RPA molecules was reduced, the binding stability was increased at any given RPA concentration. Moreover, pmRPA-S33D was not dissociated by NSMF (Figures 6A, B and S7A–D). To see the effect of multi-phosphorylated RPA32, we tested pmRPA-S4/8&S33D, in which serines 4, 8, and 33 were replaced by aspartic acid. Similar to pmRPA-S33D, the triple RPA mutant stably bound to ssDNA in the 30-nt mode and was resistant to dissociation by NSMF, suggesting that phosphorylation of S33 by ATR is sufficient to alter the RPA binding mode and that NSMF no longer affects RPA binding to ssDNA once RPA32 is phosphorylated (Figures 6C, D and S8A–D).

We also showed that phosphorylated RPA binds ssDNA in the 30-nt mode using *in vitro* RPA phosphorylation with the SV40 replication system in HEK293T cell extracts. Although the exact phosphorylated residues were not determined, the phosphorylated RPA (pRPA) exhibited similar behavior to the phosphomimetic RPA mutants in response to NSMF, supporting our suggestion that RPA32 phosphorylation promotes the stable 30-nt binding mode (Figures 6E, F and S9B–E). Compared with unphosphorylated RPA, the band shift for the pRPA–ssDNA complex was less retarded at the same concentration, indicating that phosphorylation of RPA reduces ssDNA binding. These results are consistent with a previous report showing that hyperphosphorylation of RPA decreases its ability to bind ssDNA (56). How RPA32 phosphorylation leads to the preferential formation of the 30-nt mode is not yet understood. However, it has been proposed that the phosphorylated N-terminus of RPA32 directly interacts with the basic cleft of the RPA70 DBD-F domain through electrostatic attraction, leading to a global conformational change in RPA (57). We speculate that the electrostatic interaction and conformational change suppress the unstable binding mode(s) and facilitate the 30-nt binding mode.

**Figure 7.**
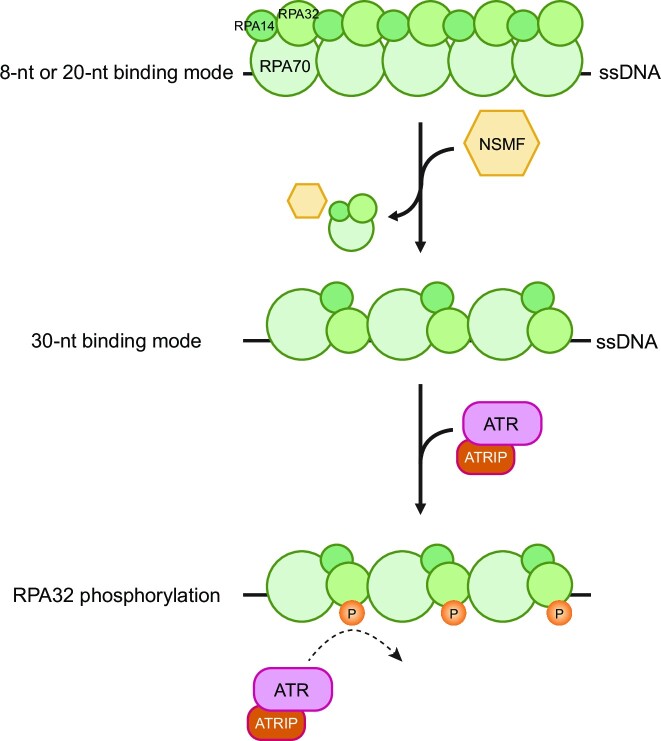
Model for the effect of NSMF on RPA phosphorylation. Immediately after ssDNA is exposed due to replication stress, RPA compactly binds to ssDNA in less stable mode(s). NSMF partially dissociates RPA from the ssDNA, and the remaining RPA molecules form a stable 30-nt binding mode, which promotes RPA phosphorylation via ATR and ATRIP.

In our laser microirradiation experiments, NSMF was recruited to DNA damage sites only at the very early stage (Figure 1A). Intriguingly, this short period of recruitment enhanced RPA phosphorylation by ATR. Thus, when RPA begins to accumulate at DNA damage sites, NSMF detaches unstably bound RPA molecules to induce the stable 30-nt binding mode, promoting RPA32 phosphorylation. This RPA phosphorylation drives the phosphorylation of other residues in RPA32 and ATR itself. Once RPA32 is phosphorylated, NSMF no longer interacts with RPA. Thus, it is no longer needed at the DNA damage sites (Figure 7).

## Supplementary Material

gkad543_Supplemental_File

## Data Availability

The data underlying this article are available in the article and in its online supplementary material.
