# Supplementary material for: Alteration of replication protein A binding mode on single-stranded DNA by NSMF potentiates RPA phosphorylation by ATR kinase
Source: Nucleic Acids Res. 2023 Jun 28;51(15):7936–50. doi: 10.1093/nar/gkad543 (PMC10450186; doi:10.1093/nar/gkad543)
Supplement: gkad543_Supplemental_File [file gkad543_supplemental_file.pdf]

Alteration of replication protein A binding mode on single-stranded DNA by NSMF potentiates RPA phosphorylation by ATR kinase

Yujin Kang[1†], Ye Gi Han[1†], Keon Woo Khim[1], Woo Gyun Choi[1], Min Kyung Ju[1], Kibeom Park[1], Kyeong Jin Shin[1], Young Chan Chae[1], Jang Hyun Choi[1*], Hongtae Kim[1,2*], and Ja Yil Lee[1,2*]

[1] Department of Biological Sciences, Ulsan National Institute of Science and Technology, Ulsan, 44919, Republic of Korea

[2] Institute of Basic Science Center for Genomic Integrity, Ulsan, 44919, Republic of Korea

† The authors are equally contributed.

* The authors are corresponding authors.

Jang Hyun Choi: janghchoi@unist.ac.kr

Hongtae Kim: khtcat@unist.ac.kr

Ja Yil Lee: biojayil@unist.ac.kr

Contents

I. Supplementary Materials and Methods

1. Cell-based experiments

1-1. Cell culture

1-2. Laser microirradiation and imaging of cells

1-3. Transfection and small interfering RNAs (siRNAs)

1-4. Plasmids

2. Immunoprecipitation

2-1. Antibodies and dilution factors

2-2. Immunoprecipitation assays

3. Protein purification

3-1. NSMF purification

3-2. Human wild-type RPA and DNA binding mutant RPA (DBM-RPA) purification

3-3. Human wild-type RPA-eGFP purification

3-4. Human RPA subunit (RPA32 and RPA14) purification

3-5. Human 7xHis wild-type RPA and 7xHis phosphomimetic RPAs (S33D and S4/8D&S33D) purification

4. DNA preparation

5. Biochemical and biophysical assays

5-1. Electrophoretic mobility shift assay (EMSA)

5-1-1. EMSA for NSMF and RPA-coated ssDNA complex

5-1-2. NSMF and various types of DNA substrates

5-2. Magnetic bead pulldown assay

5-3. Single-molecule photobleaching assay

5-4. *In vitro* phosphorylation of RPA

5-5. *In vitro* binding assay

5-6. ATR kinase assay

II. Supplementary Figures and Legends

1. Supplementary Figure S1. Interaction between NSMF and RPA at DNA damage sites

2. Supplementary Figure S2. Purification of NSMF and RPA

3. Supplementary Figure S3. Interaction between NSMF and DNA

4. Supplementary Figure S4. Effect of RPA binding defective mutant NSMF-ΔD2 on RPA32 phosphorylation and RPA binding

5. Supplementary Figure S5. Interaction between NSMF-D2 and RPA on ssDNA *in vitro*

6. Supplementary Figure S6. RPA domains that interact with NSMF

7. Supplementary Figure S7. Effect of phosphomimic RPA32-S33D on ssDNA binding

8. Supplementary Figure S8. Effect of phosphomimic RPA32-S4/8D&S33D on ssDNA binding

9. Supplementary Figure S9. Effect of *in vitro* phosphorylated RPA32 on ssDNA binding

III. Supplementary Table: oligomer list

Supplementary Materials and Methods

1. Cell-based experiments

1-1. Cell culture

HeLa and human embryonic kidney (HEK) 293T cells were purchased from American Type Culture Collection (ATCC). The cell lines were maintained in Dulbecco-modified Eagle's medium (DMEM; Invitrogen) supplemented with 10% fetal bovine serum (FBS; Millipore) and 1% penicillin/streptomycin (Gibco) at 37°C under 5% v/v CO_2. NSMF KO cell line was cultured as described previously (1). HeLa cells with stable Flag-NSMF-WT or D2 expression were obtained upon antibiotic selection with 3 μg/ml puromycin (InvivoGen) for 2 weeks. Clones were pooled into a single population to avoid clonal heterogeneity.

1-2. Laser microirradiation and imaging of cells

For laser microirradiation, HeLa cells were grown on 35 mm glass-bottom dishes (MatTek Corporation, Ashland, MA). Laser microirradiation was carried out using a Nikon A1 laser microdissection system equipped with a 37°C chamber and CO_2 module (Nikon, Tokyo, Japan). Ultraviolet A laser with 355 nm wavelength was illuminated on selected regions at 100% power for 20 iterations to induce localized DNA damage. For GFP or mCherry-tagged proteins, time-lapse images were acquired with 10 sec or 15 sec time intervals after laser microirradiation. The fluorescence intensity on each laser strip was recorded with NIS elements C software (Nikon) and analyzed with ImageJ software. The fluorescence values for >10 cells from three independent experiments were normalized to the original signal and plotted as fluorescence versus time using OriginPro (Origin Lab).

1-3. Transfection and small interfering RNAs (siRNAs)

Transient transfections with plasmid DNA and siRNAs were performed using Lipofectamine 3000 (Thermo Fisher Scientific) and Lipofectamine RNAiMAX (Thermo Fisher Scientific), respectively, according to the manufacturer's instructions. The control siRNAs described in the previous literature were used (2). The following custom siRNA sequences RPA32 #1: 5'-GGCUCCAACCAACAUUGUU-3', RPA32 #2: 5'-CCUAGUUUCACAAUCUGUU-3' were synthesized by Bioneer Inc. (South Korea).

1-4. Plasmids

The SFB-NSMF, FLAG-NSMF, GFP-NSMF, NSMF D-1, D-2, D-3, D-4, and D-5 deletion mutant, Myc-RPA32 expression plasmids were described previously (1). GFP-RPA70, RPA70 D-1, D-2, D-3, D-4, and D-5 deletion mutant expression plasmids as well as GFP-RPA32, RPA32 D-1, D-2, and D-3 deletion mutant expression plasmids were created using GFP-tagged mammalian expression vector. GFP-human RPA32 expression plasmid was created using GFP-tagged mammalian expression vector and a Myc-RPA32 expression vector. GFP-RPA32 DBM and S33D mutant expression plasmids were generated by mutagenesis using a GFP- RPA32 expression plasmid. Myc-RPA70 and Myc-RPA14 expression plasmids were created using Myc-tagged mammalian expression vector and p11d-tRPA123 plasmid. All mammalian expression plasmids were transfected using Lipofectamine^{TM} 3000 transfection reagent (Thermo Fisher Scientific) following the manufacturer's instructions.

2. Immunoprecipitation

2-1. Antibodies and dilution factors

The dilutions of various antibodies used for western blot analysis were as follows: 1:1000 for anti-NSMF (OAAN03468, Aviva Systems Biology), anti-RPA70 (A300-241A, Bethyl Laboratories, Inc.), anti-RPA32 (A300-244A, Bethyl Laboratories, Inc.), anti-RPA14 (PA5-21277, Thermo Fisher Scientific), anti-phosphoRPA32 (S33, A300-246A, Bethyl Laboratories, Inc.), anti-phophoRPA32 (S4/8, A300-244A, Bethyl Laboratories, Inc.), anti-FLAG (F3156, Sigma-Aldrich), and anti-ATR (A300-137A, Bethyl Laboratories, Inc.); 1:2000 for anti-GFP (632380, Clontech), anti-Myc (11814150001, Roche), anti-β-actin (A5316, Sigma-Aldrich), anti-α-tubulin (05-829, Millipore), and Horseradish peroxidase-conjugated secondary antibodies specific to mouse IgG (A9917, Sigma-Aldrich); 1:3000 for anti-phospho-RPA32 (S33) (A300-246A, Bethyl Laboratories, Inc.); 1:10000 for Horseradish peroxidase (HRP)-conjugated secondary antibodies specific to rabbit- (A0545, Sigma Aldrich) or mouse- (A9917, Sigma Aldrich) IgG.

2-2. Immunoprecipitation assays

For most immunoprecipitation assays except for Figure S1C and S1D, cells were washed with ice-cold phosphate buffered saline (PBS) and followed by lysis using 1% Triton X-containing lysis buffer (50 mM Tris-HCl [7.4], 150 mM NaCl, 10% Glycerol, 1 mM EDTA, 1 mM EGTA, 1 mM NaF, 1 mM Na_3VO_4, 2.5mM β-glycerophosphate, 1% Triton-X, and 1 mM $Na_4P_2O_7$ supplemented with 50 μg/ml phenylmethylsulfonyl fluoride (PMSF)) at 4°C. Lysates were cleared by centrifugation at 15,800 g at 4°C for 15 min. The supernatants were incubated with FLAG-M2 agarose (Sigma Aldrich, St. Louis, MO, USA) or primary antibodies coupled to protein G agarose (Invitrogen, CA) at 4°C. The immunoprecipitates were washed three times with lysis buffer and subjected to SDS-PAGE. Western blotting was performed with indicated antibodies and visualized using HRP-conjugated secondary antibodies and enhanced chemiluminescence (Thermo Fisher Scientific). Signals were detected using Odyssey Fc (LI-COR, Inc.).

HEK293T cells were washed with ice-cold phosphate buffered saline (PBS) and then lysed in NETN buffer (0.5% nonidet P-40, 20 mM Tris-HCl [8.0], 50 mM NaCl, 50 mM NaF, 100 μM Na_3VO_4, 1 mM dithiothreitol (DTT), and 50 μg/ml phenylmethylsulfonyl fluoride (PMSF)), and 250 units/ml benzonase (M018H, Enzynomics) and 1 mM $MgCl_2$ were added to each sample. The reactants were then incubated at 4°C for 45 min. Crude lysates were sonicated and cleared by ultracentrifugation at 15,800 g at 4°C for 10 min, and supernatants were incubated with protein A-agarose-conjugated antibody. The immunocomplexes were washed three times with NETN buffer and subjected to SDS-PAGE. Western blotting was performed using the antibodies indicated in the figures. Proteins were visualized using secondary horseradish peroxidase-conjugated antibodies (Enzo Life Sciences, New York, NY) and enhanced chemiluminescence reagent (ABC-3001, AbClon). Signals were detected using an automated imaging system (ChemiDoc™; Bio-Rad Laboratories).

3. Protein purification

All protein purification processes were performed at 4°C

3-1. NSMF purification

The gene encoding NSMF was cloned into pET19b-derived plasmid, which has MBP (maltose binding protein), PreScission cleavage site and 3xFLAG at the N-terminus and 10xHis at the C-terminus (Figure S2A). NSMF-D2 mutant was generated by a mutagenesis kit (NEB). The protein was expressed in *E. coli* (Rosetta DE3, 70954-4CN, Millipore). For NSMF purification, 8 L cells were grown at 37°C until OD_{600} reached ~ 0.6, and then the protein expression was expressed with 1 mM IPTG (Isopropyl β-D-1thiogalactopyranoside) and further incubated at 16°C overnight. The cells were harvested and resuspended in resuspension buffer (50 mM Tris-HCl [7.5], 400 mM NaCl, and 0.01% Tween-20) with 1xprotease inhibitor (Halt, 78439, Thermo Fisher Scientific). All the following processes were performed at 4°C. The cells were lysed using sonication, and the cell lysates were ultracentrifuged at 50,000 g for 1 hr. The clarified lysate was mixed with 4 ml of amylose resin (S6651, NEB), which was pre-equilibrated with amylose buffer (50 mM Tris-HCl [7.5], 400 mM NaCl, 1 mM EDTA, 1 mM DTT, and 0.01% Tween-20). After 2-hr rotation, the mixture was packaged into an empty column (bed volume: 2 ml). The amylose resin was washed with amylose buffer, and MBP was cleaved out by overnight incubation of 40 units/ml PreScission protease (27-0843-01, Cytiva) in amylose buffer. Then the eluant was further purified by gel filtration with Superdex200 10/300 GL (17-5175-01, Cytiva) in size exclusion buffer (50 mM Tris-HCl [7.5], 200 mM NaCl, 1 mM EDTA, 1 mM DTT, and 0.01% Tween-20). Fractions containing NSMF were pooled and then dialyzed against 2 L storage buffer (10 mM Tris-HCl [7.5], 200 mM NaCl, 1 mM EDTA, 1 mM DTT, 0.01% Tween-20, and 10% glycerol). The purified NSMF was stored at -80°C after snap-freezing by liquid N_2.

3-2. Human wild-type RPA and DNA binding mutant RPA (DBM-RPA) purification

Human wild-type RPA with all three subunits (RPA70, RPA32, and RPA14) was purified as described in the previous literature (3). Briefly, the plasmid having all RPA subunits was kindly provided by Patrick Sung (UT Health San Antonio) and was transformed into *E. coli* BL21(DE3)pLysS strain (C6060-03, Thermo Fisher Scientific). 6 L cells in 2xLB media were grown at 37°C until OD_{600} ~ 0.6. The protein expression was induced by 1 mM IPTG and further incubated at 16°C overnight. Cells were harvested and resuspended in resuspension buffer (100 mM Tris-HCl [7.5], 600 mM KCl, 10 mM EDTA, 0.01% Igepal, 1 mM DTT, and 1 mM benzamidine). The cells were then lysed by sonication and spun down at 100,000 g at 4°C for 40 min. The clarified lysate was bound to 30 mL bed volume of Affi-gel blue column (153-7302, Bio-Rad) and washed with T-800 (800 mM NaCl in T-buffer (25 mM Tris-HCl [7.5], 0.5 mM EDTA, 1 mM DTT, and 10% glycerol)). RPA was eluted by salt gradient from T-100 buffer (100 mM NaCl in T-buffer) to 2.5 M NaSCN buffer (2.5 M NaSCN in T-buffer). The pooled fractions were diluted up to 100 mM of salt concentration with T-buffer and further bound to 8 mL bed volume of Macrohydroxyapatite column (157-0040, Bio-Rad). Then RPA was eluted by salt gradient from T-100 buffer to 0.4 M phosphate buffer (0.4 M KH_2PO_4 in T-buffer). Pooled fractions were diluted up to 100 mM of salt concentration with T-buffer. Finally, RPA was purified by using 1 ml Q-HP column (17-1153-01, Cytiva) with salt gradient from T-100 buffer to T-1000 buffer (1 M NaCl in T-buffer). Pooled fractions were concentrated by Amicon MWCO 30 K (UFC9030, Millipore) up to 5 mg/ml and then diluted up to 40 mM NaCl with T-buffer. The protein was stored at -80°C after being snap-frozen by liquid N_2.

DNA binding mutant of RPA32 (DBM-RPA), where tryptophan 107 (W107) and phenylalanine 135 (F135) were both replaced by alanine, were also purified as the same procedure as the

wild-type.

3-3. Human wild-type RPA-eGFP purification

Human wild-type RPA-eGFP (RPA-eGFP), in which eGFP is tagged at the C-terminus of RPA70, was purified as follows. The plasmid containing 6xHis-tagged RPA-eGFP was transformed into *E. coli* BL21(DE3)pLysS. 6 L cells in 2xLB media were grown at 37°C until OD_{600} ~ 0.6. The protein expression was induced by 1 mM IPTG and further incubated at 16°C overnight. Cells were then pelleted and resuspended in T-100 buffer with 1xprotease inhibitor (Halt, 78439, Thermo Fisher Scientific). Cells were lysed by sonication, and the lysate was clarified by centrifugation at 100,000 g for 1 hr at 4°C. The clarified lysate was loaded onto 30 mL bed volume of Affi-gel blue column (153-7302, Bio-Rad) that was equilibrated with T-100 buffer. The column was washed with T-100 buffer and further with T-800 buffer. Proteins were eluted by salt gradient from T-100 buffer to 2.5 M NaSCN buffer. The fractions containing RPA-eGFP were pooled and then diluted 10 times by Talon wash buffer (50 mM HEPES-NaOH [7.4], 250 mM NaCl, and 20 mM Imidazole) The protein was loaded onto 15 ml bed volume of Talon column (635670, TaKaRa) that was equilibrated with Talon wash buffer. After the column was washed with Talon wash buffer, RPA-eGFP was eluted with Talon elution buffer (Talon wash buffer supplemented with 500 mM imidazole). RPA-eGFP fractions were pooled and dialyzed against storage buffer (50 mM Tris-HCl [8.0], 40 mM NaCl, 0.5 mM EDTA, 1 mM DTT, and 10% glycerol). The final stock was stored at -80°C.

3-4. Human RPA subunit (RPA32 and RPA14) purification

Each subunit of human RPA (RPA32 and RPA14) was subcloned in pTXB1-derived plasmid having 6xHis at the N-terminus. Each plasmid was transformed into *E. coli* BL21(DE3) strain. 8 L cells were grown in LB media at 37°C until OD_{600} reached ~ 0.6, and then the protein expression was induced with 1 mM IPTG, and cells were further incubated at 16°C overnight. The cells were harvested and resuspended in T-100 buffer with 1xprotease inhibitor (Halt, 78439, Thermo Fisher Scientific). The cells were lysed using sonication, and the lysates were ultracentrifuged at 100,000 g for 70 min. The clarified lysates were loaded to 15 ml bed volume of HisPur™ Ni-NTA resin **(**88222, Thermo Fisher Scientific), which was pre-equilibrated with Ni-wash buffer (T-100 buffer supplemented with 20 mM imidazole). After Ni-NTA resin was washed with 5xcolumn volume (CV) of Ni-wash buffer, proteins were eluted with Ni-elution buffer (T-100 buffer supplemented with 500 mM imidazole). The fractions containing a RPA subunit were pooled and then diluted 10 times by T-buffer. Proteins were then purified by 1 ml Q column (HiTrap Q HP, 17115301, Cytiva) by salt gradient from T-100 buffer to T-1000 buffer. Fractions containing RPA32 or RPA14 were pooled and then dialyzed against 2 L storage buffer (50 mM Tris-HCl [8.0], 40 mM NaCl, 0.5 mM EDTA, 1 mM DTT, and 10% glycerol). Each purified RPA subunit was stored in -80°C after being snap-frozen by liquid N_2.

3-5. Human 7xHis wild-type RPA and 7xHis phosphomimetic RPAs (S33D and S4/8D&S33D) purification

7xHis wild-type RPA was purified for *in vitro* phosphorylation in pET11d-derived plasmid. The plasmid was transformed into *E. coli* BL21(DE3) strain. 8 L cells were grown in LB media at 37°C until OD_{600} reached ~ 0.6, and then the protein was expressed with 1 mM IPTG, and cells were further incubated at 16°C overnight. The cells were then harvested and resuspended in T-100 buffer with 1xprotease inhibitor (Halt, 78439, Thermo Fisher Scientific). The cells

were lysed using sonication, and the lysates were ultracentrifuged at 100,000 g for 1 hr. The clarified lysates were loaded to 15 ml bed volume of HisPur™ Ni-NTA resin (88222, Thermo Fisher Scientific), which was pre-equilibrated with Ni-wash buffer (T-100 buffer with 20 mM imidazole). After Ni-NTA resin was washed with 5xCV of Ni-wash buffer, proteins were eluted with Ni-elution buffer (T-100 buffer with 500 mM imidazole). The fractions containing 7xHis RPA or 7xHis pmRPA were pooled and further purified by gel filtration with Superdex200 10/300 GL (17-5175-01, Cytiva) in T-100 buffer. Fractions containing 7xHis RPA or 7xHis pmRPA were pooled and then dialyzed against 2 L storage buffer (50 mM Tris-HCl [8.0], 40 mM NaCl, 0.5 mM EDTA, 1 mM DTT, and 10% glycerol). The purified protein was stored at -80°C after being snap-frozen by liquid N_2.

7xHis phosphomimetic RPA that had either single S33D mutation or triple S4/8D and S33D mutations in RPA32 was purified by the same protocol as 7xHis wild-type RPA.

4. DNA preparation

Oligomers were all synthesized (Bioneer, South Korea). All DNA constructs were prepared by following the previous protocol (4). Oligomers were mixed at equi-molar ratio in 10 mM Tris-HCl [7.5] and 100 mM NaCl. For annealing, the mixtures were heated at 95°C for 10 min and slowly cooled to 23°C.

5. Biochemical and biophysical assays

5-1. Electrophoretic mobility shift assay (EMSA)

5-1-1. EMSA for NSMF and RPA-coated ssDNA complex

All reactions were performed in reaction buffer (50 mM Tris-HCl [7.5], 50 mM NaCl, 1 mM DTT, and 0.01% Tween-20) at 23°C. 10 nM of fluorescently-labeled single-stranded oligomer (14, 30, 60, or 91 nts) was incubated with WT RPA, DBM-RPA32, phosphomimetic RPA (pmRPA), or *in vitro* phosphorylated RPA (pRPA) at different concentrations for 20 min. To test the effect of NSMF on RPA-coated ssDNA, NSMF or NSMF-ΔD2 was titrated to the RPA-ssDNA complexes and incubated for 30 min. In addition, to test the effect of NSMF on RPA binding to ssDNA, 200 nM NSMF or NSMF-D2 was pre-incubated with ssDNA, and then RPA was added at different concentrations. The reactions were analyzed by 4.5 ~ 7% non-denaturing polyacrylamide gel electrophoresis (PAGE) in TBE buffer at 4°C. The gel was imaged by Typhoon RGB (Cytiva).

5-1-2. NSMF and various types of DNA substrates

All reactions were performed in reaction buffer (50 mM Tris-HCl [7.5], 50 mM NaCl, 1 mM DTT, and 0.01% Tween-20) at 23°C. 10 nM various types of Cy5-labeled DNA substrates, such as duplex, ssDNA, primer-template junction (PTJ), gap, bubble, D-loop, R-loop, or fork (Y-shape), were incubated with NSMF at different concentrations in the reaction buffer for 30 min (Supplementary Table). The reactants were analyzed by running 1.5% agarose gel in 1xTBE buffer at 4°C, and the gel was imaged by Typhoon RGB (Cytiva). To estimate the dissociation equilibrium constant (K_d), we fitted the bound fraction graph with hyperbola function

$Bound\ fraction = A[NSMF]/(K_d + [NSMF])$.

5-2. Magnetic bead pulldown assay

300 nM of biotinylated 80-nt ssDNA was conjugated to 5 ul of streptavidin-coated magnetic beads (Dynabeads M-280, Invitrogen) in 20 mM Tris-HCl [8.0] and 100 mM NaCl at 23°C for 2 hrs. After unbound DNA was removed, 1.2 uM RPA was incubated with the ssDNA-decorated magnetic beads at 23°C for 50 min. Free RPA proteins were removed, and 20 nM NSMF or NSMF-D2 was incubated with the beads in reaction buffer for 30 min. Then the beads were pulled down and only supernatant was taken. The protein portion bound to ssDNA was eluted by boiling the beads at 95°C for 3 min. The supernatant and eluant were analyzed via western blot using an anti-FLAG antibody (A2220, Sigma) and anti-RPA32 antibody (A300-244A, Thermo Fisher Scientific).

5-3. Single-molecule photobleaching assay

The single-molecule photobleaching assay was conducted as described before (5). All reactions were performed at 23°C in reaction buffer. 0.3 nM biotinylated 14-, 30-, 60-, or 80-nt ssDNA was anchored on a streptavidin-coated slide surface (Supplementary Table 1). After unbound DNA was washed out, 0.75 nM RPA-eGFP was injected into the flowcell and incubated for 20 min. Free RPA proteins were washed out using 200 ul of reaction buffer containing 1xgloxy and 1.6% glucose three times, and 100 nM NSMF or NSMF-D2 mutant was incubated for 30 min. After residual NSMF was flushed out with reaction buffer containing 1xgloxy and 1.6% glucose, the fluorescence signal of RPA-eGFP was collected under the illumination of a 488 nm laser until almost all eGFP fluorescent puncta were photobleached. Fluorescence signals were imaged by NIS-element (Nikon). Images were analyzed by a customized software smCamera, which was kindly provided by Professor Kyung Suk Lee at Kongju National University. Each fluorescent punctum was fitted with a 2D Gaussian function, and then the fluorescence trajectories were extracted. The photobleaching steps were counted from each fluorescence intensity trajectory.

5-4. In vitro phosphorylation of RPA

In vitro phosphorylation was performed to generate phosphorylated RPA using methods previously described (6,7) with minute modification. HEK293T cells were resuspended with ice-cold resuspension buffer (25 mM Tris-HCl [8.0], 100 mM NaCl, and 10% Glycerol) supplemented with 1xprotease inhibitor cocktail (P2714, Sigma), 1xphosphatase inhibitors cocktails (P5726 and P0044, Sigma), and 1xPMSF (10837091001, Merck) and lysed by sonication. The lysates were clarified by centrifugation at 22,000 g and 4°C for 30 min. The concentration of clarified lysates was measured by Bradford assay. *In vitro* phosphorylation reaction was conducted by mixing 2 ng/µl of pcDNA3, 2 ng/µl of M13mp18 ssDNA (N4040S, NEB), 5 mg/ml of HEK293T cell lysates prepared above, and 500 nM of purified 7xHis-wild-type RPA in 1xphosphorylation buffer (40 mM HEPES [7.5], 8 mM $MgCl_2$, 3 mM ATP, and 0.5 mM DTT). The reactants were incubated at 37°C for 2 hrs. *In vitro* RPA phosphorylation was confirmed by western blot using anti-RPA32, anti-phosphoRPA32 (S4/8), and anti-phosphoRPA32 (S33) and phosphatase assay. The in vitro phosphorylated RPA (pRPA) was incubated with 400 units of λ-PPase (P0753, NEB) containing 1xNEBuffer pack for Protein MetalloPhosphatases (PMP) supplemented with 1 mM $MnCl_2$ at 30°C for 1 hr. After the reaction was stopped by the addition of protein sample buffer, the reactant was subjected to

SDS-PAGE and western-blotted. Then the pRPA was purified using Ni-NTA resin (HisPur™ Ni-NTA Resin. 88222, Thermo Fisher Scientific). 1 ml of Ni-NTA resin was equilibrated in Ni-wash buffer (50 mM HEPES [7.5], 250 mM NaCl, and 20 mM imidazole). 750 ul of phosphorylated RPA was gently mixed with the resin and incubated in a cold room for 1 hr. After being washed with Ni-wash buffer three times, the resin was resuspended in Ni-elution buffer (50 mM HEPES [7.5], 250 mM NaCl, and 500 mM imidazole) followed by 10 min incubation. The proteins were eluted by taking supernatant after centrifugation (800 g for 1 min). The buffer was exchanged into storage buffer (25 mM Tris_HCl [7.5], 40 mM NaCl, 1 mM DTT, 0.5 mM EDTA, and 10% glycerol) during concentration with Amicon (MWCO 30 kDa).

5-5. In vitro binding assay

Purified hRPA and eGFP-tagged NSMF WT or D-2 were used for in vitro binding assay. RPA and eGFP-NSMF WT or D-2 were incubated in NETN at 4°C for 1 hr and then anti-GFP-coupled protein G agarose (Pierce Protein G Agarose, 20399, Thermo Fisher Scientific) was added and additionally incubated at 4°C for 1 hr. The immunoprecipitates were washed three times with NETN and subjected to western blotting.

5-6. ATR kinase assay

For ATR kinase assay, 10-nt, 20-nt, and 30-nt ssDNA oligomers consisting of only thymines (dT_{10}, dT_{20}, and dT_{30}) were synthesized (Bioneer, South Korea) (Supplementary Table 1). Human ATR protein was prepared from HeLa cells, which were treated with 2 mM hydroxyurea (HU) for 16 hrs. The cells were washed with ice-cold 1xPBS and then lysed in IP buffer (50 mM HEPES [7.5], 150 mM NaCl, 1% Triton X-100, and 50 µg/ml PMSF) at 4°C for 10 min. Crude lysates were cleared by centrifugation at 20,000 g at 4°C for 5 min, the supernatants were incubated with proteinA-agarose (CA-PRI-0005, Repligen) that was conjugated ATR antibodies (A300-137A, Bethyl Laborartories). The immunoprecipitated ATR kinases were washed three times in IP buffer.

To test the effect of 30-nt binding mode on RPA32 phosphorylation, 75 nM RPA was incubated with 30 nM ssDNA (dT_{10}, dT_{20}, or dT_{30}) in ATR kinase buffer (20 mM HEPES [8.0], 10 mM $MgCl_2$, 2 mM DTT, and 0.1 mM ATP) at 23°C for 20 min. Then, 25 ul of the reactant was added to the immunoprecipitated ATR kinases and incubated at 30°C for 50 min. After the reaction, samples were separated by SDS PAGE and analyzed by western blotting with indicated antibodies.

To test the effect of NSMF on RPA32 phosphorylation, excessive RPA (150 nM) was incubated with 10 nM of 91-nt ssDNA in ATR kinase buffer at 23°C for 20 min, and then 80 nM NSMF was added followed by 30 min incubation. 25 ul of the reactant was added to the immunoprecipitated ATR kinases and incubated at 30°C for 30 min. After the reactions, all samples were separated by SDS PAGE and analyzed by western blotting with indicated antibodies.

II. Supplementary Figures and Legends

1. Supplementary Figure S1. Interaction between NSMF and RPA at DNA damage sites

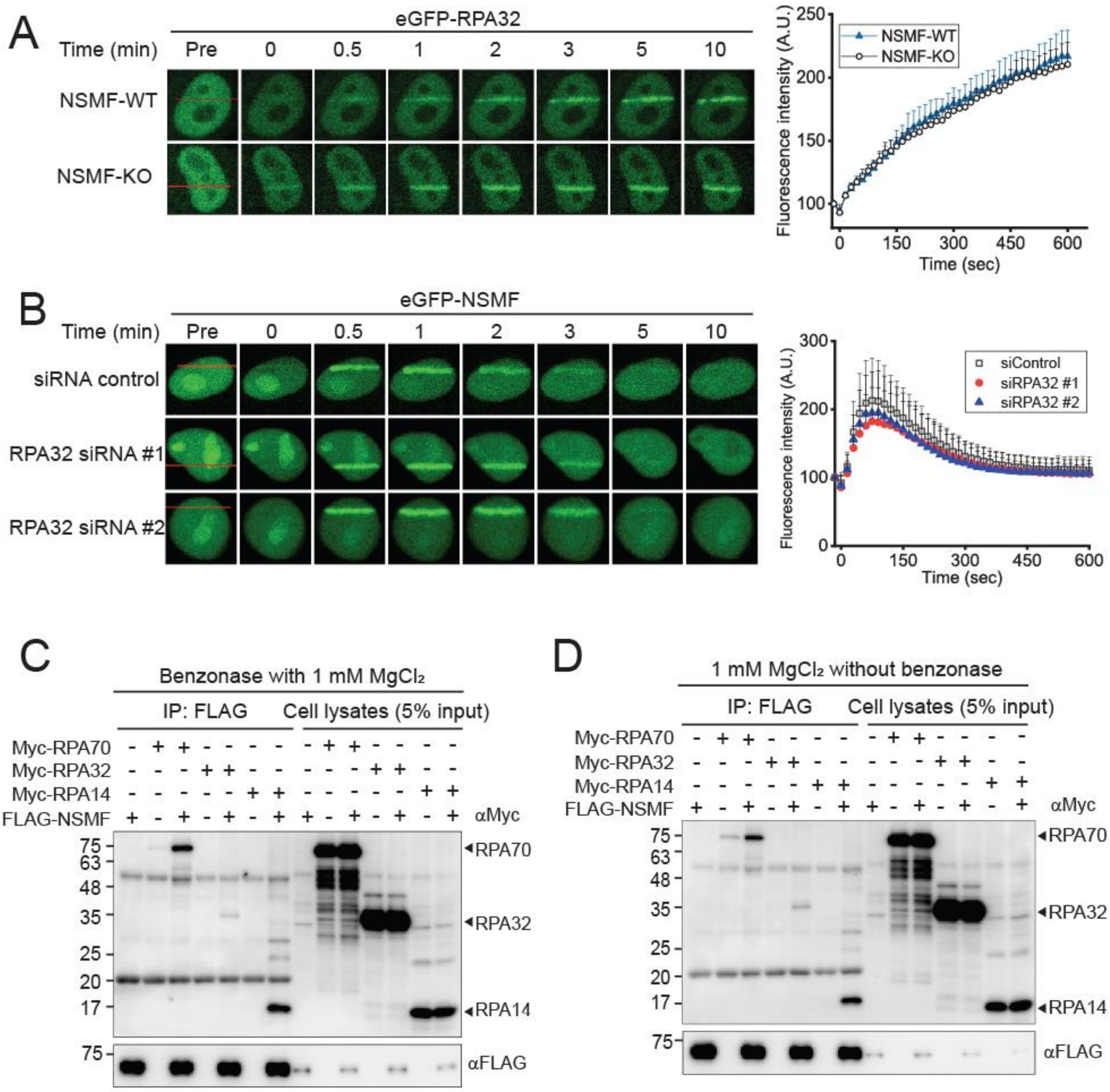

(A) Laser microirradiation experiments for NSMF-WT or NSMF-KO HeLa cells, which were transfected with eGFP-RPA. Left: after 24 hr-incubation, the cells were laser microirradiated, and the recruitment of eGFP-RPA to DNA damage was examined by live cell imaging. Red lines indicate the laser-irradiated sites. Right: the quantified relative intensity of eGFP at the damage as a function of time. For each condition, we tested greater than 10 cells and repeated the same experiments three times for one cell. The error bar represents a standard error in triplicate. (B) Laser microirradiation experiments for NSMF-WT or NSMF-KD HeLa cells, which were transfected with a control or RPA32 siRNAs. After 24 hr-incubation, the cells were transfected with eGFP-NSMF and incubated for another 24 hrs. Left: the cells were then laser microirradiated, and the recruitment of eGFP-NSMF to DNA lesions was examined by live cell

imaging. Red lines indicate the laser-irradiated sites. Right: the relative intensity of eGFP is quantified. For each condition, we tested greater than 10 cells and repeated the same experiments three times for one cell. The error bar represents a standard error in triplicate. (C-D) The interaction between NSMF and each RPA subunit using IP in an overexpression system. HEK293T cells were transfected with indicated plasmids for 24 hrs and lysed (C) with or (D) without benzonase. Cell lysates were immunoprecipitated with anti-FLAG agarose beads and analyzed by western blotting with an anti-Myc antibody (αMyc).

Supplementary Figure S2. Purification of NSMF and RPA

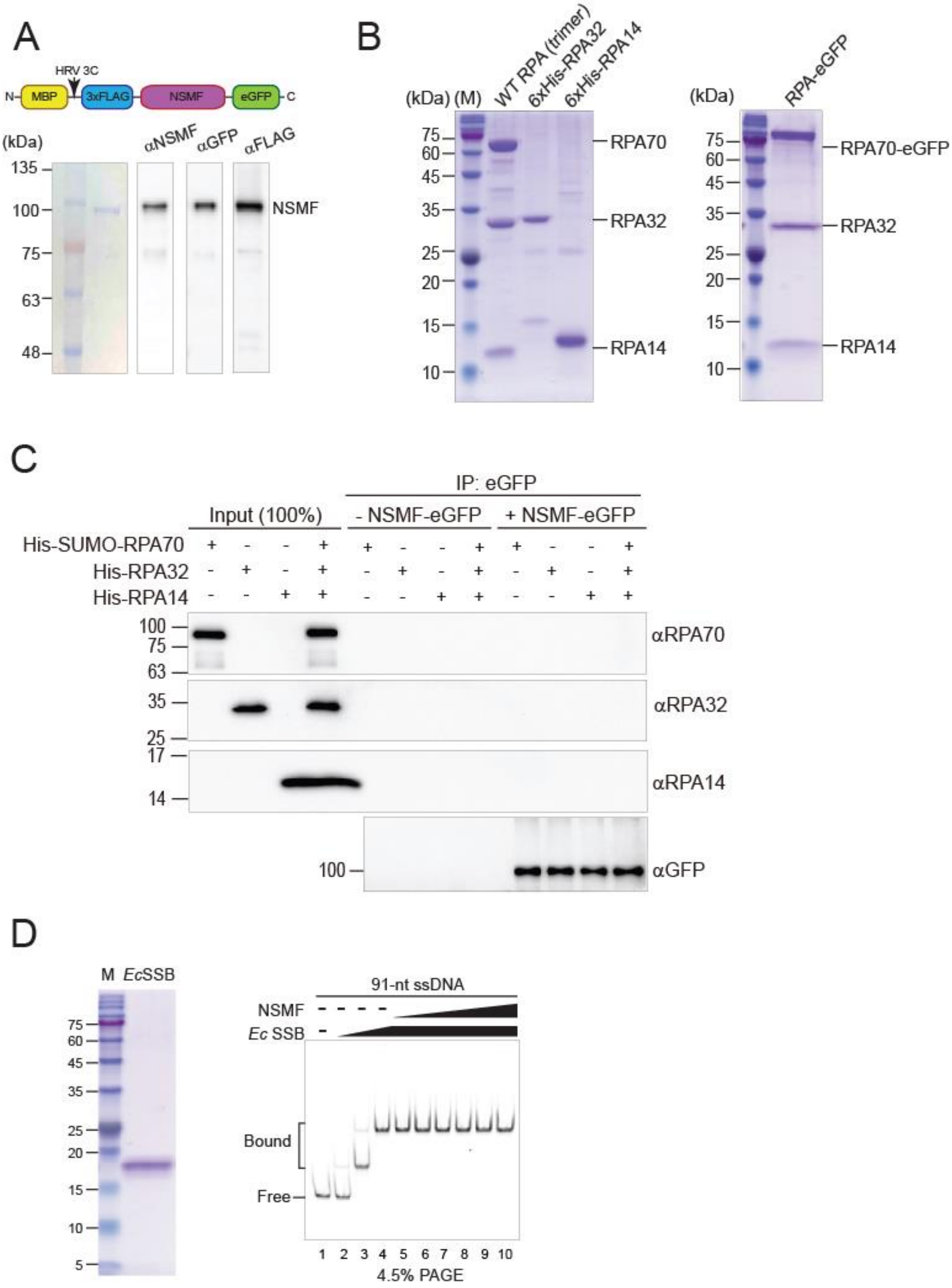

(A) Purification of full-length 3xFLAG-NSMF-eGFP. Top: schematic of NSMF construct. Maltose binding protein (MBP) that is eliminated by HRV 3C protease and 3xFLAG is placed at N-terminus, and eGFP is tagged at C-terminus. Bottom: 10% SDS PAGE of purified full-length NSMF (~90 kDa) and western blotting with antibodies for NSMF (αNSMF), GFP (αGFP), and FLAG (αFLAG). (B) SDS PAGE for purified wild-type (WT) RPA trimer, RPA32 subunit, RPA14 subunit, and RPA-eGFP (eGFP is tagged at RPA70 subunit). (C) *In vitro* IP for the interaction between NSMF-eGFP and each RPA subunit. RPA70 subunit was purchased (MBS967200, MyBioSource) and RPA32 and RPA14 subunits were purified. Each RPA subunit and NSMF-eGFP were incubated and then immunoprecipitated using anti-GFP antibody. The immunoprecipitants were analyzed by western blotting with indicated antibodies. (D) 12% SDS PAGE of *E. coli* SSB (EcSSB) (S3917, Sigma Aldrich). EMSA for NSMF and *E. coli* SSB (EcSSB)-ssDNA complex with 91-nt ssDNA. EcSSB (0, 10, 50, and 150 nM) was titrated to 10 nM ssDNA, and then NSMF was titrated (0, 10, 20, 40, 80, 140, and 200 nM) at 150 nM EcSSB.

Supplementary Figure S3. Interaction between NSMF and DNA

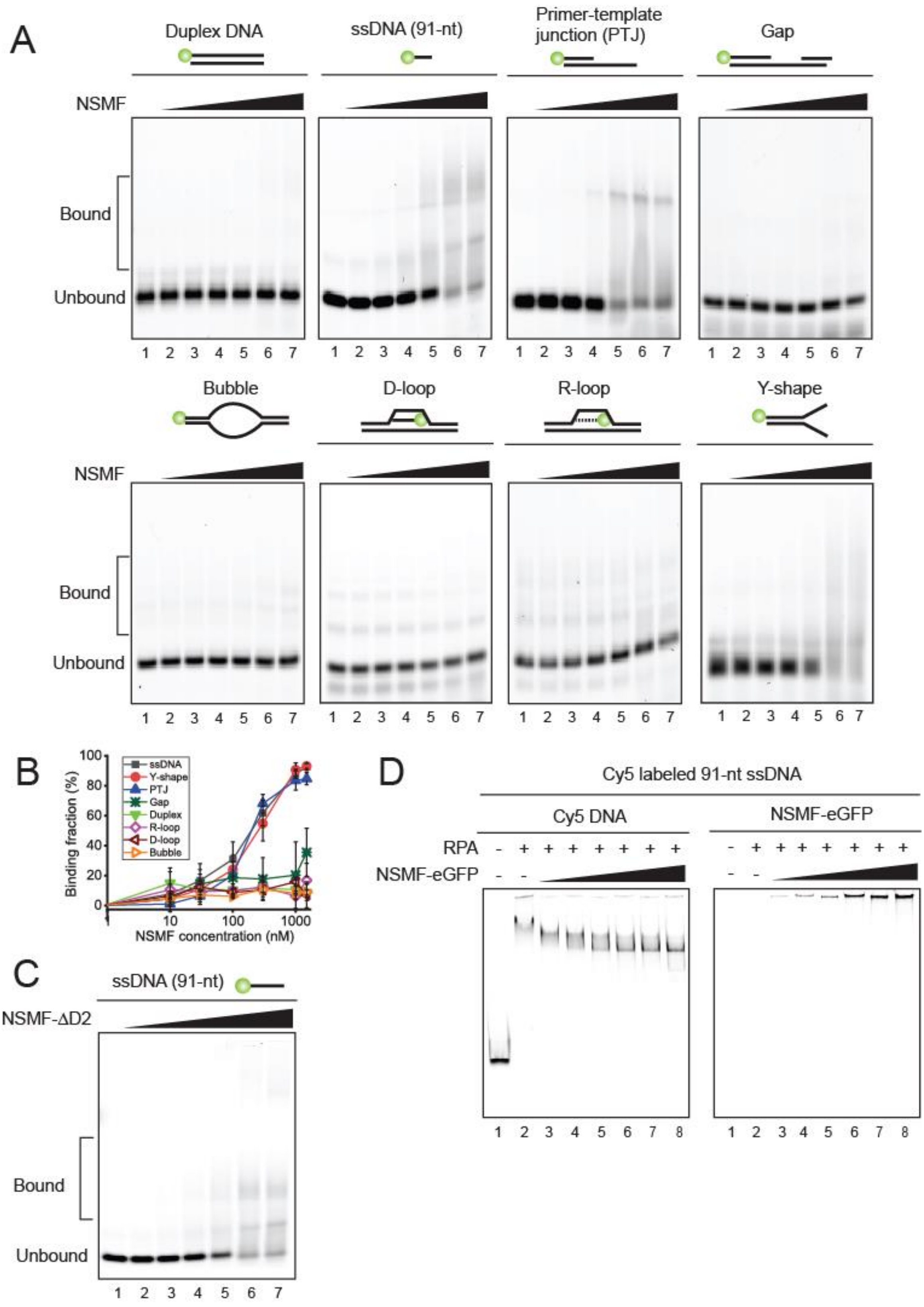

(A) 1.5% agarose gel electrophoresis for EMSA with NSMF and various nucleic acid constructs labeled with Cy5: homoduplex, ssDNA (91-nt), primer-template junction (PTJ), gap, bubble, D-loop, R-loop, and fork (Y-shape). NSMF (0, 10, 30, 100, 300, 1000, and 3000 nM) was titrated to 10 nM of each construct. (B) Quantification of EMSA data in (A), gray filled square: ssDNA, red filled circle: Y-shape, blue filled triangle: PTJ, dark green asterisk: 50-nt gap, green filled inverted triangle: duplex, purple blank diamond: R-loop, brown blank left triangle: D-loop, and orange blank right triangle: bubble. (C) 1.5% agarose gel electrophoresis for EMSA with NSMF-D2 and 91-nt ssDNA. Quantification is shown in Figure 4E. (D) EMSA of NSMF-eGFP with for Cy5-labeled ssDNA to which RPA bound. NSMF-eGFP is stuck in the well without overlapping with ssDNA, indicating that NSMF does not co-bind to the RPA-ssDNA complex.

Supplementary Figure S4. Effect of RPA binding defective mutant NSMF-D2 on RPA32 phosphorylation and RPA binding

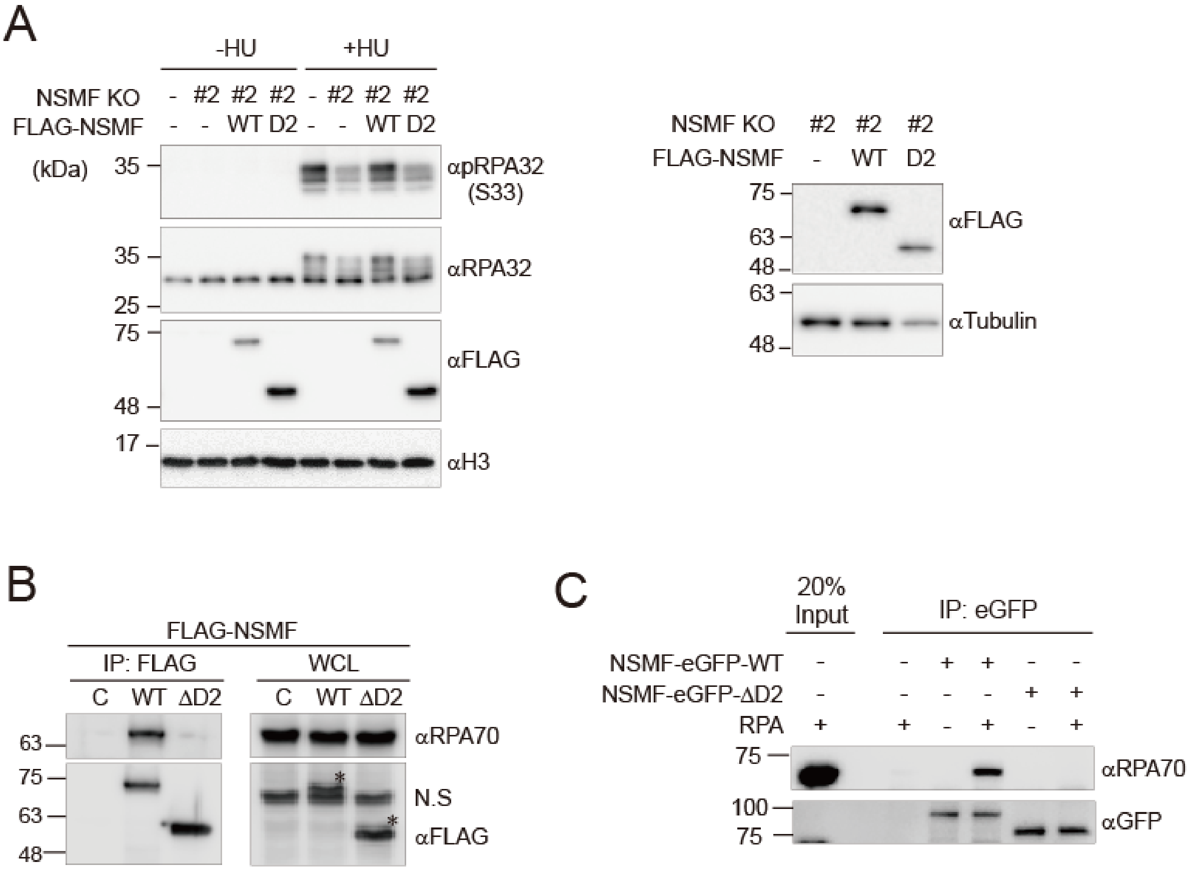

(A) (Left) Chromatin fraction analysis for NSMF-D2 effect on RPA32 phosphorylation under replication stress. (Right) NSMF expression in NSMF KO HeLa cells that express FLAG-NSMF-WT and D2. (B) Interaction between RPA70 and either NSMF WT or NSMF D2 using immunoprecipitation from HeLa cells that were transfected with FLAG-NSMF WT or D2. Asterisks denote bands for NMSF WT and D2. (C) *In vitro* binding assay using RPA70 and either NSMF-eGFP WT or D2 that were all purified.

Supplementary Figure S5. Interaction between NSMF-D2 and RPA on ssDNA *in vitro*

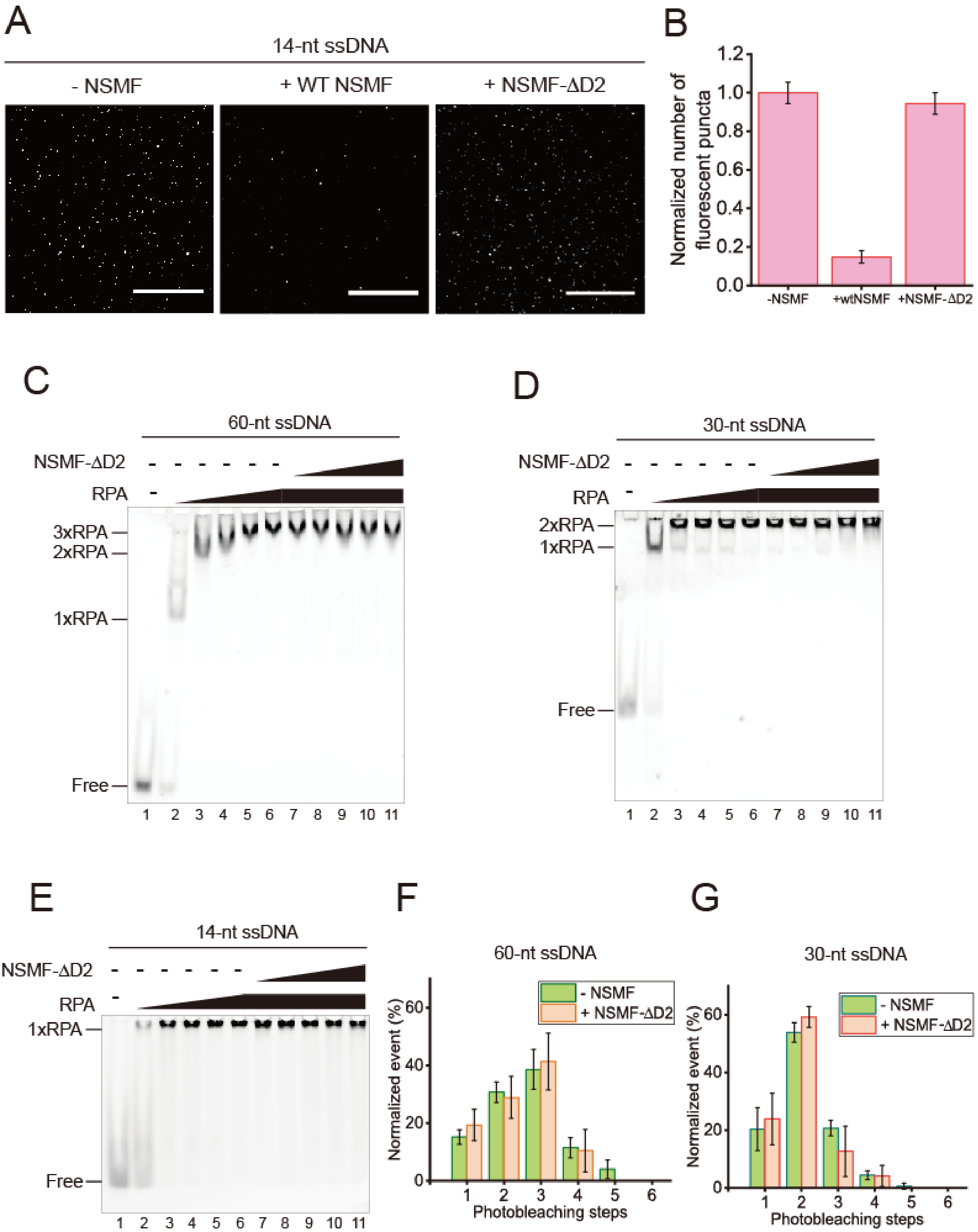

(A) Fluorescence images of RPA-eGFP bound to 14-nt ssDNA. The fluorescent puncta indicate that RPA-eGFP molecules bind to individual 14-nt ssDNA molecules in the presence of no NSMF (left), WT NSMF (middle), and NSMF-D2 (right). The scale bar represents 30 μm. (B)

Histograms for the normalized number of RPA-eGFP molecules bound to 14-nt ssDNA in (A). Error bars were obtained from the standard deviation in triplicate. (C-E) EMSA for NSMF-D2 and RPA with (C) 60-nt, (D) 30-nt, and (E) 14-nt ssDNA. RPA (0, 25, 75, 100, 125, and 150 nM) was titrated to 10 nM of each ssDNA substrate, and then NSMF-D2 was titrated (0, 10, 20, 40, 80, and 140) at 150 nM RPA. (F-G) Histograms of photobleaching steps of RPA-eGFP bound to (F) 60-nt and (G) 30-nt ssDNA. The distribution of photobleaching steps of RPA-eGFP in the presence of NSMF-D2 (orange) is similar to that in the absence of NSMF (green). The number of analyzed molecules was greater than 150 for each experiment. Error bars were obtained from the standard deviation in triplicate.

Supplementary Figure S6. RPA domains that interact with NSMF

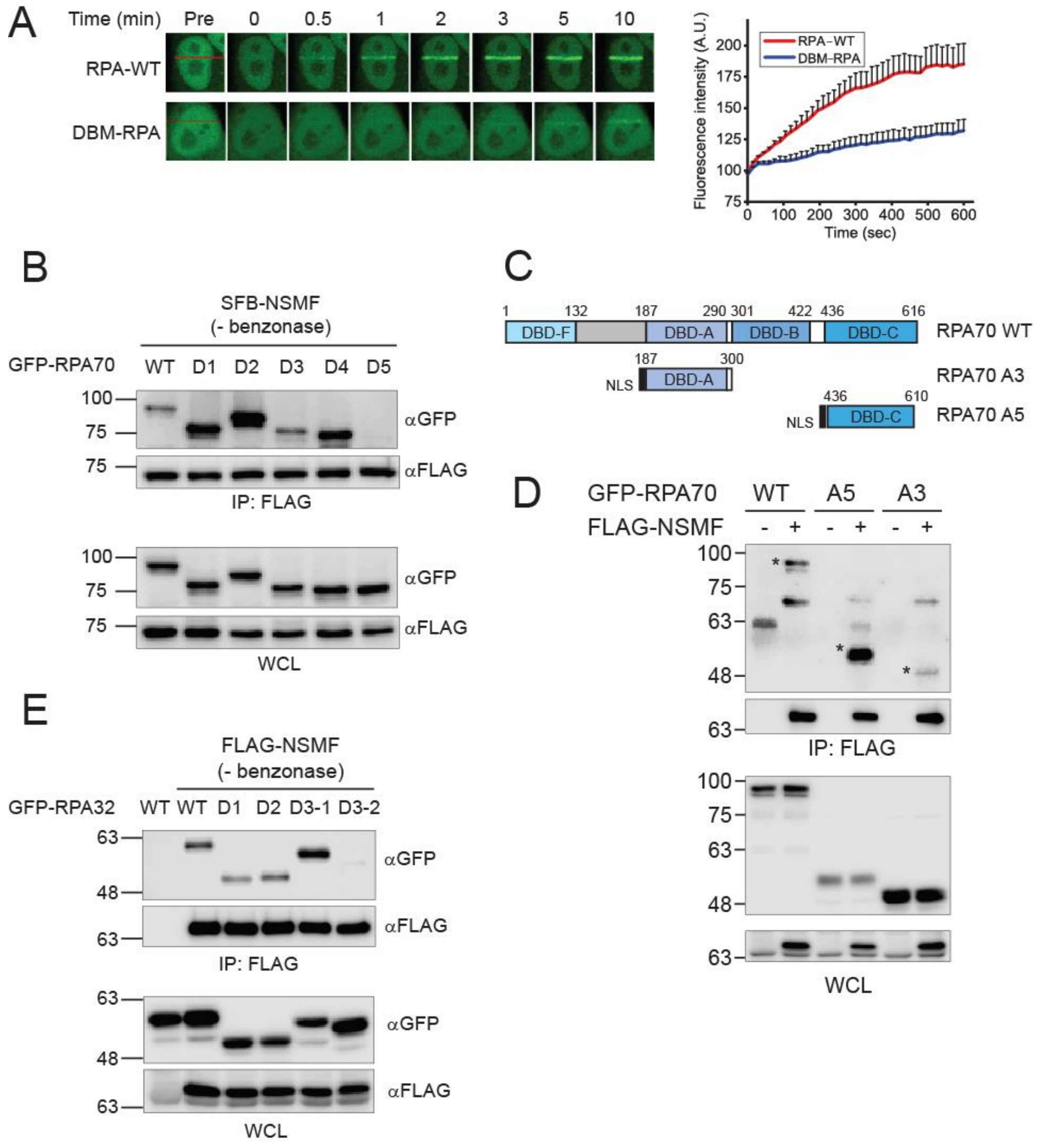

(A) Laser microirradiation experiments for GFP-RPA (WT) or GFP-DBM-RPA in HeLa cells. DBM-RPA was weakly recruited to DNA damage sites. Left: fluorescence images of a laser-irradiated cell as a function of time. The red lines indicate the laser-irradiated sites. Right: quantified relative fluorescence intensity of eGFP on laser stripes. The initial fluorescent intensity on damage was set as 100 for each cell, and the recruitment kinetics was plotted. The intensity is averaged for more than 30 cells under each condition, and the data represent the mean + SEM in triplicate. (B) IP assays between NSMF and RPA70 deletion mutants without benzonase. (C) Domain constructs of add-back mutants of RPA70. (D) IP assays between NSMF and RPA70 add-back mutants with benzonase. (E) IP assays between NSMF and RPA32 deletion mutants without benzonase.

Supplementary Figure S7. Effect of phosphomimic RPA32-S33D on ssDNA binding

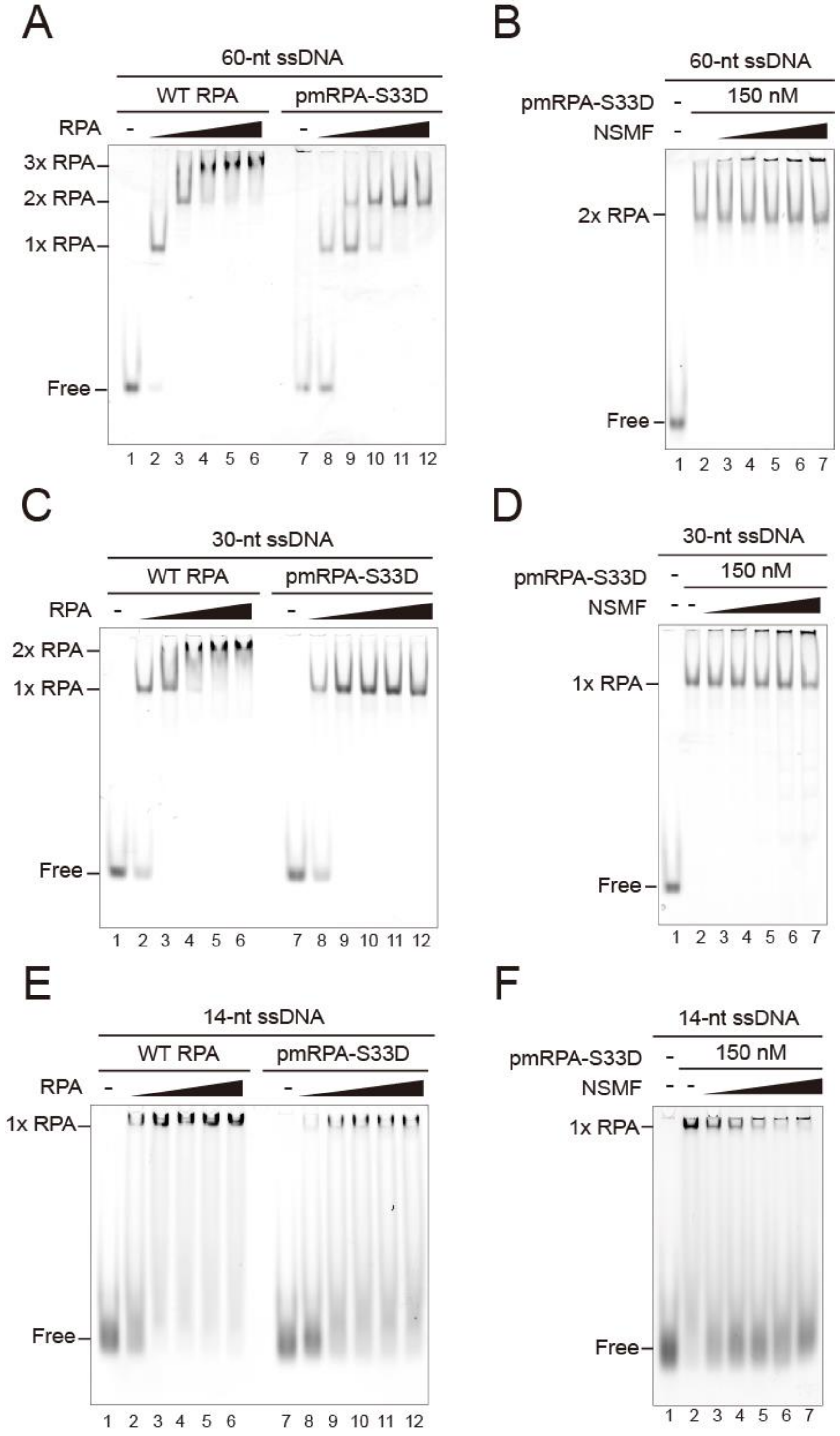

(A, C, and E) EMSA for WT RPA and pmRPA-S33D (0, 25, 75, 100, 125, and 150 nM) to compare their binding to 60-nt (A), 30-nt (C), and 14-nt (E) ssDNA. (B, D, and F) EMSA for NSMF effect on pmRPA-S33D. NSMF (0, 20, 40, 80, 140, and 200 nM) was titrated at 150 nM pmRPA-S33D that was pre-incubated with 10 nM 60-nt (B), 30-nt (D), and 14-nt (F) ssDNA.

Supplementary Figure S8. Effect of phosphomimic RPA32-S4/8D&S33D on ssDNA binding

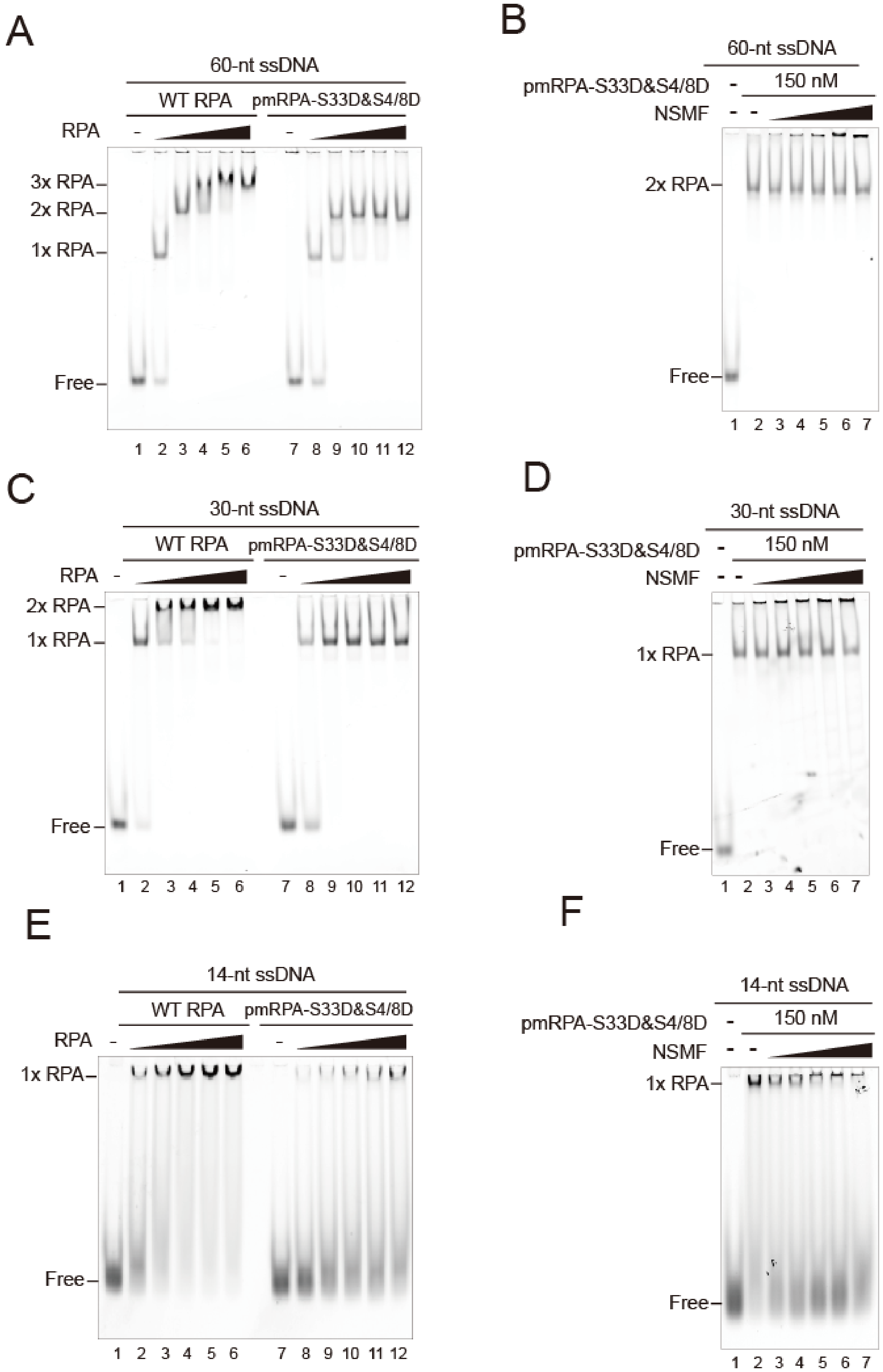

(A, C, and E) EMSA for WT RPA and pmRPA-S4/8D&S33D (0, 25, 75, 100, 125, and 150 nM) to compare their binding to 60-nt (A), 30-nt (C), and 14-nt (E) ssDNA. (B, D, and F) EMSA for NSMF effect on pmRPA-S4/8D&S33D. NSMF (0, 20, 40, 80, 140, and 200 nM) was titrated at 150 nM pmRPA-S4/8D&S33D that was pre-incubated with 10 nM 60-nt (B), 30-nt (D), and 14-nt (F) ssDNA.

Supplementary Figure S9. Effect of *in vitro* phosphorylated RPA32 on ssDNA binding

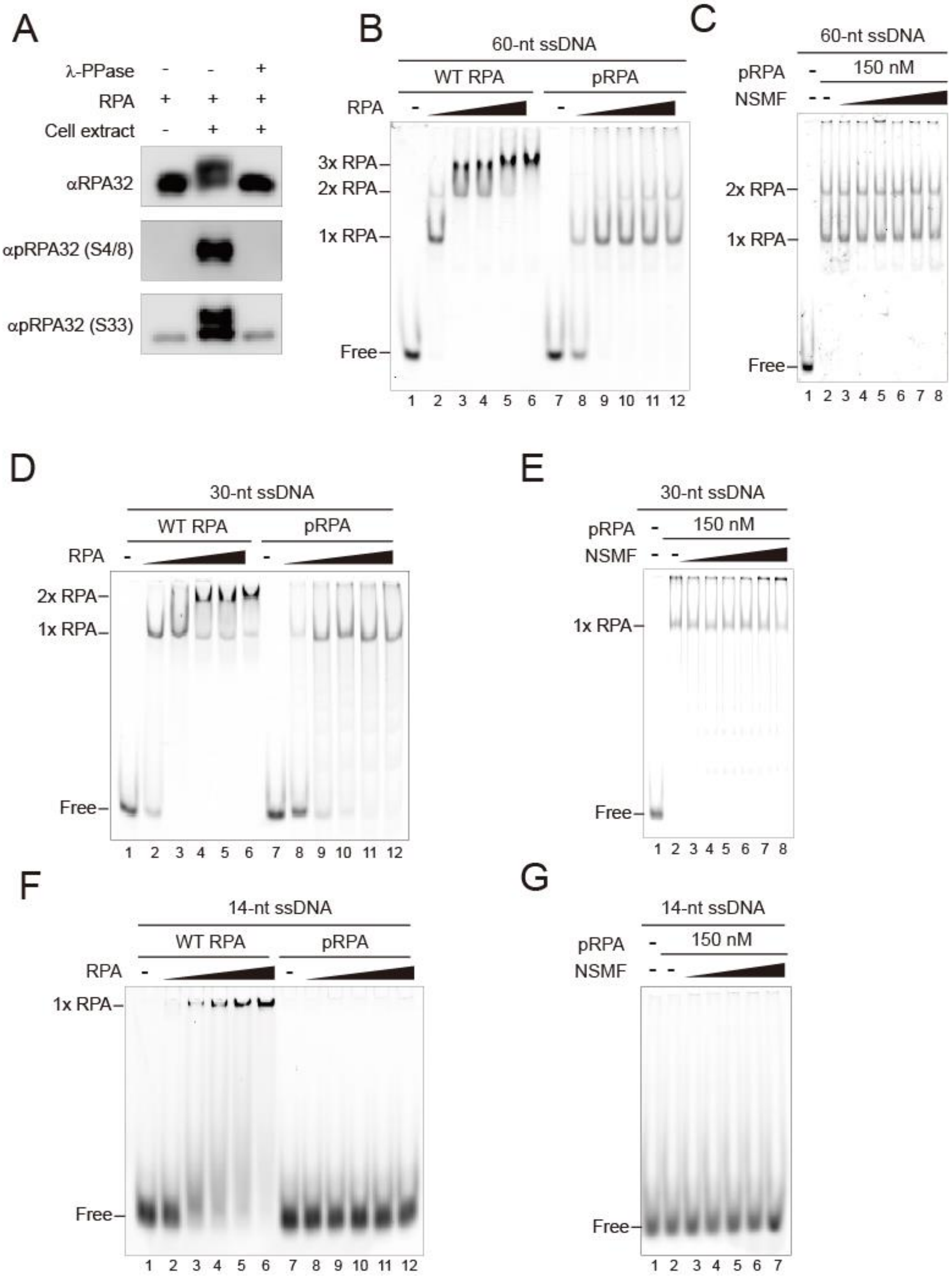

(A) *In vitro* phosphorylation of RPA (pRPA) with HEK293T cell extracts. The phosphorylation at S4/8 or S33 of RPA32 was detected by western blotting with indicated antibodies. The phosphorylation was also confirmed by λ-phosphatase (λ-PPase) treatment. (B,D, and F)

EMSA for WT RPA and pRPA (0, 25, 75, 100, 125, and 150 nM) to compare their binding to 60-nt (B), 30-nt (D), and 14-nt (F) ssDNA. (C and E) EMSA for NSMF effect on pRPA. NSMF (0, 10, 20, 40, 80, 140, and 200 nM) was titrated at 150 nM pRPA that was pre-incubated with 10 nM 60-nt (C) and 30-nt (E) ssDNA. (G) EMSA for NSMF effect on pRPA. NSMF (0, 20, 40, 80, 140, and 200 nM) was titrated at 150 nM pRPA that was pre-incubated with 14-nt ssDNA.

Supplementary Table: Oligomer List

Name	Sequence (5' → 3')
91-nt ssDNA	[Cy5] GCCAGGGACGAGGTGAACCTGCAGGTGGGCTTTTTTT TTTTTTTTTTTTTTTTTTTTTTTTTGGTAGAATTCGGCAG CGTCATGCGACGGC
60-nt ssDNA	[Cy5] TATTCATTGTTTTACCTATTGTACCTTATTCTTAATTCA TTTTACTAATCATTTACGAGT
Cy5-30-nt ssDNA	[Cy5] TTTTTTTTTTTTTTTTTTTTTTTTTTTTTT
30-nt ssDNA	TTTTTTTTTTTTTTTTTTTTTTTTTTTTTT
20-nt ssDNA	TTTTTTTTTTTTTTTTTTTT
14-nt ssDNA	[Cy5] TTTTTTTTTTTTTT
10-nt ssDNA	TTTTTTTTTT
Biotinylated 80-nt ssDNA	[Biotin] ATACCGGTAGATATCCGACCTAGCCAACAGTACGGTC TTGCGATCACACTTCTAGCTAGTGCTCGATCGATCGAT CACAG
Biotinylated 60-nt ssDNA	[Biotin] TATTCATTGTTTTACCTATTGTACCTTATTCTTAATTCA TTTTACTAATCATTTACGAGT
Biotinylated 30-nt ssDNA	[Biotin] TTTTTTTTTTTTTTTTTTTTTTTTTTTTTT
Biotinylated 14-nt ssDNA	[Biotin] TTTTTTTTTTTTTT
Oligo1	[Cy3] GCCAGGGACGAGGTGAACCTGCAGGTGGGCGGCTACT ACTTAGATGTCATCCGAGGCTTATTGGTAGAATTCGG CAGCGTCATGCGACGGC
Oligo2	GCCGTCGCATGACGCTGCCGAATTCTACCACGCGATT CATACCTGTCGTGCCAGCTGCTTTGCCCACCTGCAGGT TCACCTCGTCCCTGGC
RNA oligo	[Cy5] GCAGCUGGCACGACAGGUAUGAAUC
D-loop oligo	[Cy5] GCAGCTGGCACGACAGGTATGAATC
Oligo2 comp	[Cy5] GCCAGGGACGAGGTGAACCTGCAGGTGGGCAAAGCA GCTGGCACGACAGGTATGAATCGCGTGGTAGAATTCG GCAGCGTCATGCGACGGC
PTJ oligo	TGG CGA CGG CAG CGA GGC AGG TCA GGA ATT CTG ATC
PTJ oligo comp	GAT CAG AAT TCC TGA CCT [Cy3]
Y-shape oligo	[Cy5] GCCAGGGACGAGGTGAACCTGCAGGTGGGCGGC TACTACTTTTTTTTTTTTTTTTTTTTTTTTTTTTTTTTTTT TTTTT
Y-shape oligo comp	TTTTTTTTTTTTTTTTTTTTTTTTTTTTTTTTTTTTTTTAG TAGTAGCCGCCCACCTGCAGGTTCACCTCGTCCC

	TGGC
Gap oligo1	AGAATCCCGGTGCCGAGGCCGCTCAATTGGTCGTAGA CA
Gap oligo2	GCCAAGGGGATTACTCCCTAGTCTCCAGGCACGTGTC AGATATATACATC
Gap oligo comp	CTCGAGGATGTATATATCTGACACGTGCCTGGAGACT AGGGAGTAATCCCCTTGGCGGTTAAAACGCGGGGAC AGCGCGTACGTGCGTTTAAGCGGTGCTAGAGCTGTCT ACGACCAATTGAGCGGCCTCGGCACCGGGATTCT

* R-loop DNA: Oligo1, Oligo2, and RNA oligo were hybridized

* D-loop DNA: Oligo1, Oligo2, and D-loop oligo were hybridized

* Duplex DNA: Oligo2 and Oligo2 comp were hybridized

* Bubble DNA: Oligo1 and Oligo2 were hybridized

* PTJ DNA: PTJ oligo and PTJ oligo comp were hybridized

* Y-shape DNA: Y-shape oligo and Y-shape oligo comp were hybridized

* Gap DNA: Gap oligo1, Gap oligo2, and Gap oligo comp were hybridized

References

1. Ju, M.K., Shin, K.J., Lee, J.R., Khim, K.W., E, A.L., Ra, J.S., Kim, B.G., Jo, H.S., Yoon, J.H., Kim, T.M. *et al.* (2021) NSMF promotes the replication stress-induced DNA damage response for genome maintenance. *Nucleic Acids Res*, **49**, 5605-5622.
2. Kim, H., Huang, J. and Chen, J.J. (2007) CCDC98 is a BRCA1-BRCT domain-binding protein involved in the DNA damage response. *Nat Struct Mol Biol*, **14**, 710-715.
3. Henricksen, L.A., Umbricht, C.B. and Wold, M.S. (1994) Recombinant replication protein A: expression, complex formation, and functional characterization. *J Biol Chem*, **269**, 11121-11132.
4. Kang, H.J., Cheon, N.Y., Park, H., Jeong, G.W., Ye, B.J., Yoo, E.J., Lee, J.H., Hur, J.H., Lee, E.A., Kim, H. *et al.* (2021) TonEBP recognizes R-loops and initiates m6A RNA methylation for R-loop resolution. *Nucleic Acids Res*, **49**, 269-284.
5. Kang, Y., Cho, C., Lee, K.S., Song, J.J. and Lee, J.Y. (2021) Single-Molecule Imaging Reveals the Mechanism Underlying Histone Loading of Schizosaccharomyces pombe AAA+ ATPase Abo1. *Mol Cells*, **44**, 79-87.
6. Soniat, M.M., Myler, L.R., Kuo, H.C., Paull, T.T. and Finkelstein, I.J. (2019) RPA Phosphorylation Inhibits DNA Resection. *Molecular Cell*, **75**, 145-+.
7. Fotedar, R. and Roberts, J.M. (1992) Cell-Cycle Regulated Phosphorylation of Rpa-32 Occurs within the Replication Initiation Complex. *Embo Journal*, **11**, 2177-2187.
